# Hyperarousal transdiagnostically dissected: different dimensions characterize mood, anxiety, insomnia, posttraumatic stress and attention deficit hyperactivity disorders

**DOI:** 10.1016/j.eclinm.2026.103810

**Published:** 2026-03-12

**Authors:** Tom Bresser, Siemon C. de Lange, Lara Rösler, Tessa F. Blanken, Sophie van der Sluis, Eus J.W. Van Someren

**Affiliations:** a Department of Sleep and Cognition, Netherlands Institute for Neuroscience (NIN), an Institute of the Royal Netherlands Academy of Arts and Sciences, Amsterdam, the Netherlands; b Department of Complex Trait Genetics, Center for Neurogenomics and Cognitive Research, Amsterdam Neuroscience, Vrije Universiteit Amsterdam, Amsterdam, the Netherlands; c Department of Psychology, University of Amsterdam, the Netherlands; d Department of Child and Adolescent Psychiatry and Psychology, Section Complex Trait Genetics, Amsterdam Neuroscience, Vrije Universiteit Medical Center, Amsterdam University Medical Center, Amsterdam, the Netherlands; e Department of Integrative Neurophysiology, Center for Neurogenomics and Cognitive Research (CNCR), Amsterdam Neuroscience, Vrije Universiteit Amsterdam, the Netherlands; f Department of Psychiatry, Amsterdam Public Health Research Institute and Amsterdam Neuroscience Research Institute, Amsterdam UMC, Vrije Universiteit, the Netherlands

**Keywords:** Hyperarousal, Transdiagnostic, Insomnia, Anxiety disorders, Posttraumatic stress disorder, Major depressive disorder

## Abstract

**Background:**

Hyperarousal is a common symptom key to the severity of insomnia-, depression-, anxiety-, posttraumatic stress- and attention deficit/hyperactivity disorders. Hyperarousal however remained a loosely defined construct assessed with different questionnaires in different disorders. Here we addressed the unresolved question whether hyperarousal may be one common transdiagnostic construct or rather has multiple, possibly disorder-specific, dimensions.

**Methods:**

In this cohort study, participants were recruited through media and from the Netherlands Sleep Registry between Dec 2023 and June 2024. We included 467 adults (mean age 58.3 years [range 21–89]; 77.6% female) with a wide range of psychiatric diagnoses and severities who completed all hyperarousal questionnaires and disorder symptom severity scales (Insomnia Severity Index, Rapid Measurement Toolkit-20, and ADHD Self-Report Scale). Factor analyses evaluated potential dimensions in hyperarousal assessed with 221 items from 18 questionnaires. Multiple regression models were used to reveal profiles of the most relevant hyperarousal dimensions associated with the symptom severity of insomnia disorder, major depressive disorder, generalized anxiety disorder, social anxiety disorder, panic disorder, posttraumatic stress disorder and attention deficit/hyperactivity disorders. We used 27 selected items representing the seven dimensions of hyperarousal to create the Transdiagnostic Hyperarousal Dimensions Questionnaire (THDQ). A second sample was recruited between March 2025 and April 2025 for confirmation and validation (n = 592; mean age 61.0 years [range 19–89]; 65.2% female), who completed the THDQ. To examine the possibility to estimate hyperarousal dimension factor scores using available UK Biobank items, we calculated the polychoric correlation between the 27 selected items and 22 UK Biobank items in 467 adults.

**Findings:**

Exploratory factor analysis identified 7 dimensions, explaining 50.2% of the variance and representing anxious-, somatic-, sensitive-, sleep-related-, irritable-, vigilant- and sudomotor hyperarousal. Multiple regression models showed that hyperarousal dimensions differentially correlated with the severity of insomnia, major depression, anxiety, panic, posttraumatic stress and attention deficit/hyperactivity disorder symptoms (standardized beta-coefficients = −0.10 to 0.70). We next developed and validated a 27-item THDQ, reliably assessing each dimension (CFI = 0.92, RMSEA = 0.05, Cronbach's alpha = 0.90). Finally, we showed that UK Biobank items can estimate anxious, irritable and sleep-related hyperarousal (r = 0.75–0.85).

**Interpretation:**

Distinguishing different hyperarousal dimensions, enabled by the THDQ, can propel our understanding of hyperarousal to provide clues for better treatment of multiple mental disorders.

**Funding:**

10.13039/501100000781European Research Council (ERC) and 10.13039/501100001826ZonMw (partnership between Care Research Netherlands and Dutch Research Council).

**Research in context**

**Evidence before this study**

A PubMed search conducted Sept 2025 using the terms “transdiagnostic” and “hyperarousal” revealed 19 results. While hyperarousal is a shared core symptom in multiple mental disorders, no previous study addressed the unresolved question whether hyperarousal may be one common transdiagnostic construct or rather has multiple, possibly disorder-specific, dimensions.

**Added value of this study**

This study discovered seven different dimensions of hyperarousal and provides a concise instrument to assess them transdiagnostically in clinical practice as well as in studies including those utilizing the large UK Biobank cohort.

**Implications of all the available evidence**

The provided concise instrument to assess all dimensions opens fast and reliable investigations of transdiagnostic hyperarousal and the underlying pathology.

## Introduction

Mental disorders affect 38–53% of the people at least once during their lifetime. At least a third will suffer from two disorders or more.^1^, ^2^, ^3^, ^4^ The most prevalent mental disorder categories are the combined anxiety disorders and insomnia disorder. Together these two mental disorder categories account for half of the global mental health burden.^3^ Since the disorders often occur together or sequentially, studying transdiagnostic factors that link them could be pivotal for understanding mechanisms and improving treatment strategies. Their key linking symptom is *hyperarousal*, which is a core symptom of not only insomnia disorder (ID) and anxiety disorders, but also of posttraumatic stress disorder (PTSD), attention deficit hyperactivity disorder (ADHD) and some manifestations of Major Depressive Disorder (MDD).^5^, ^6^, ^7^, ^8^, ^9^, ^10^ Despite the clinical relevance of hyperarousal, the lack of a transdiagnostic instrument to assess it in large follow-up cohort studies like the UK Biobank impedes mechanistic understanding.

Hyperarousal is a loosely defined concept, covering cognitive, emotional, and somatic experiences of tension and distress. Depending on the disorder of their primary interest, scholars and clinicians have focused on different manifestations of what they consider to be key representations of hyperarousal in that disorder. For example, in anxiety disorders, hyperarousal commonly refers to experiences of worry, distress or tension.^11^ In PTSD, the concept of hyperarousal includes angry outbursts, being hyperalert and jumpy, and trouble with sleep.^12^ In insomnia disorder, hyperarousal typically refers to increased cognitive, emotional and physiological complaints mostly occurring prior to sleep,^6^ even though experienced hyperarousal is actually worse after sleep.^13^

Hyperarousal can be assessed with several questionnaires, most of which target specific manifestations of hyperarousal. In anxiety disorders, and especially panic disorder (PD), somatic symptoms of hyperarousal also called ‘anxious arousal’ have been the focus of attention. While the Beck Anxiety Index focuses on such somatic complaints –e.g. ‘shaky’ and ‘heart pounding’–,^14^ the anxiety subscale of the Hospital Anxiety and Depression Scale (HADS) queries more cognitive hyperarousal—‘I feel tense or wound up’ and ‘Worrying thoughts go through my mind’–.^15^ In ID, researchers commonly use the Pre-Sleep Arousal Scale (PSAS) to capture hyperarousal occurring prior to bedtime,^16^ or the Hyperarousal Scale (HS) developed to capture behaviors considered characteristic for insomnia.^17^ It has remained unclear, however, whether different measures of hyperarousal are truly distinguishable in different disorders or reflect a shared hyperarousal construct.

A study in over 250,000 participants of the Million Veteran Program suggested that the hyperarousal symptom cluster of PTSD may act as a transdiagnostic factor that links the genetic and phenotypic relationships between PTSD, anxiety, MDD and neuroticism.^18^ Moreover, hyperarousal is the unifying factor of different theoretical models on insomnia,^6^ and insomnia shows strong genetic correlations with anxiety, depression and neuroticism.^19^ Insomnia in turn is a common symptom of PTSD^11^ with pre-deployment hyperarousal as strongest predictor for post-deployment insomnia symptoms.^20^ While based on different questionnaires, these findings suggest that hyperarousal is a transdiagnostic symptom linking insomnia-, anxiety- and PTSD. Given the apparent key importance of hyperarousal in the severity of mental disorders, their link, and their transgression to comorbidity, it is unfortunate that no previous study compared hyperarousal manifestations within the same individuals across disorders to address to what extent hyperarousal could be a transdiagnostically unifying construct.

The current study aimed to facilitate the transdiagnostic understanding and assessment of hyperarousal. We first queried an international panel of experts, representing multiple disorders characterized by hyperarousal, about the questionnaires they advised to include. We implemented the total of 221 suggested questions for online assessment in a Citizen Science approach in people with a wide range of mental health issues or vulnerabilities. In addition to the hyperarousal-related items, participants completed screeners to assess the severity of symptoms characteristic of seven mental disorders. They moreover completed a selection of UK Biobank items considered possibly relevant as indicator measures for hyperarousal. Data of 467 participants who completed all questions were factor-analyzed to reveal the dimensional structure of hyperarousal. We next evaluated how the severity of the different hyperarousal dimensions linked to the severity of symptoms of ID, MDD, generalized anxiety disorder (GAD), social anxiety disorder (SAD), PD, PTSD and ADHD. Subsequently, to facilitate concise assessment, we selected high-loading and specific items for each hyperarousal dimension and validated the resulting 27-item Transdiagnostic Hyperarousal Dimensions Questionnaire (THDQ).

Finally, given the mentioned importance of studying hyperarousal transdiagnostically in large cohorts, we identified which of the dimensions could be estimated sufficiently reliable from existing items in the UK Biobank. While the UK Biobank did not include a dedicated validated hyperarousal questionnaire, some items scattered over different questionnaires resemble items of measures for hyperarousal. Given our previous experience identifying a valid and valuable indicator measure for insomnia,^21^ we evaluated whether hyperarousal can be estimated from these items.

## Methods

### Study design and participants

Participants were recruited through media advertisement and through the website and newsletter of the Netherlands Sleep Registry (NSR, www.slaapregister.nl). The NSR cohort was added to the media advertisements as a strategic recruitment choice because, next to good sleepers, about half of the participants report insomnia complaints. Since across mental disorders only very few people do not report insomnia, we expected this approach to attract a wide variety of mental disorders. The success of the approach is evident from Table S1: 42% were ever diagnosed with a mental disorder, of whom 22% had a current diagnosis. Importantly, there was no oversampling on disordered sleep. Of the people with a current psychiatric diagnosis, 40% also had a current sleep disorder diagnosis, which is identical to the reported prevalence of sleep disorders in psychiatric outpatients.^22^ The addition of NSR to our other recruitment strategy thus did not create bias towards sleep-disordered participants. Inclusion criteria required participants to be aged 18 years or older. Between December 2023 and June 2024, a total of 866 individuals started the assessment, which contained multiple modules to be completed at their own pace, allowing multiple days. Of them, 467 completed all hyperarousal questionnaires and disorder symptom severity scales, making their data suitable for the exploratory factor analysis and subsequent analyses. Demographic data, which were missing in 8 participants, are provided in Table S2. Demographic data and symptom severity of multiple mental disorders of were similar in participants with incomplete questionnaire data (see Table S3). Between March 2025 and April 2025, after completing the exploratory factor analysis and developing the THDQ based on the results, a second sample of 592 people was recruited for confirmatory factor analysis and validation. A subset of 315 people participated in both samples, allowing for analyses of consistency between the original very extensive assessment and the THDQ. Questionnaire data collection through the Netherlands Sleep Registry was approved by the Medical Ethical Committee of the Academic Medical Center of the University of Amsterdam (29 September 2009, 09.17.1396) and the Central Committee on Research Involving Human Subjects (CCMO, 14 December 2011, CCMO11.1813/GK/jt), The Hague, the Netherlands. All participants provided digital informed consent.

### Procedures

#### Data

In the first assessment round, participants completed online questionnaires comprising three categories: (1) an extensive set of 221 questions from 18 questionnaires compiled to assess different aspects of hyperarousal; (2) three questionnaires to assess the diagnostic probability and severity of multiple mental disorders; and (3) a set of 56 UK Biobank questions selected on their estimated relevance to hyperarousal, to develop UK Biobank indicator measures for the hyperarousal dimensions. Demographics were also assessed, or were already available for existing subscribers of the NSR newsletter (n = 271). Years of education count the years beyond elementary school. Income was surveyed using 7 categories and was used as a proxy-measure for socioeconomic status. Race was assessed using self-report on the origin of the biological parents. In the second assessment round, participants only completed the newly developed 27-item THDQ.

#### Hyperarousal measures

An international panel, comprising 42 experts on different mental disorders were contacted based on a screening of literature and their subsequent recommendations for other experts, along with individuals with lived experience, was invited by email to recommend (sub)scales, items and topics relevant for assessing hyperarousal. Of them, 26 (62%) responded. No effort was made to reach consensus because the aim was diversity rather than agreement. Contributors mentioned two somewhat related concepts which they were not sure of being directly relevant for hyperarousal: catatonia and memory. These concepts were not further explored for available questionnaires to include. All other recommendations were included in the list and complemented by internet searches on assessment of hyperarousal. As a guiding principle, we included (1) all items from scales primarily assessing hyperarousal and (2) all items from subscales intended to assess hyperarousal within a broader questionnaire. This approach allowed us to evaluate hyperarousal with 221 items commonly used in scales validated to estimate the severity of the symptom in various disorders (see Table S4 for an overview). Some (sub-) scales and items represent generic measures, others distinguish specific dimensions (e.g. cognitive versus somatic hyperarousal) or types of context-based arousal (e.g. tension experienced from traumatic or shameful experiences, and hyperarousal at bedtime). Responses were coded 1 – n, maintaining the original scale range (12 dichotomous items, 91 four-level Likert items and 118 five-level Likert-type items).

#### Assessment of diagnostic probabilities and symptom severity

We adapted the mental health conditions assessment from the UK Biobank (field ID: 29000) to query self-reported current diagnoses, lifetime diagnoses and if diagnoses were confirmed by a clinician or other health care professional (see Table S1). In addition, the likely diagnostic presence of mental disorders and the severity of their symptoms were estimated using screeners: the Insomnia Severity Index^23^ (ISI, 7 items, cut-point ≥10) for insomnia disorder; the Rapid Measurement Toolkit-20^24^ (RMT20) for MDD (4 items, cut-point ≥13), GAD (4 items, cut-point ≥11), SAD (4 items, cut-point ≥12), PD (4 items, cut-point ≥9) and PTSD (4 items, cut-point ≥8); the 6-item adult ADHD self-report scale^25^ (ASRS, cut-point ≥4) for attention deficit/hyperactivity disorder (ADHD).

#### UK Biobank

We moreover aimed to evaluate the possibility of studying hyperarousal dimensions in the large and rich dataset of the UK Biobank. Next to the Eysenck Neuroticism Questionnaire (12 items) already included based on expert panel suggestions, we selected 44 more UK Biobank items that we considered likely to capture aspects of hyperarousal based on their similarity with any of the items suggested by the expert panel. The 56 items were related to sleep, anxiety (including GAD-7), worry, depressive mood (PHQ-9), neuroticism (Eysenck Neuroticism 12) and PTSD (PCL-S) (see Table S5). After omitting items with excessive missing values resulting from conditioning on other items, we selected 22 items for estimating hyperarousal dimensions from UK Biobank items (see Table S20).

### Statistical analysis

All analyses were implemented in R version 4.3.3. Table S6 shows the versions of the used packages. Prior to the exploratory factor analysis, we estimated polychoric correlations between the 221 hyperarousal items using the R package ‘psych’ with setting smooth = TRUE, global = FALSE and correcting pairwise zero counts by replacing them with 0.1 as a correction for continuity. Next, we applied the Kaiser-Meyer Olkin test to assess sampling adequacy, the proportion of variance that might be common variance, and kept items with a Measure of Sampling Adequacy value of 0.7 or higher. To obtain a meaningful factor structure and avoid multicollinearity issues we reduced the number of items by excluding items that were too similar to other included items. To keep items that capture unique variance, we applied a data driven stepwise selection procedure^26^ to include, from item pairs with correlations exceeding first |0.9| and subsequently lower by steps of −0.05, the item with the lowest absolute median correlation with all other items. We stopped at r > |0.5| because we reached ∼100 selected items which we deemed a workable number of items for the exploratory factor analysis (EFA). If two highly correlating items had a similar absolute median correlation with other items, we selected the most lasting or trait-like (rather than state-like) item. For example, items querying the last month were preferred over items querying the past two weeks. The current focus was thus on stable predisposing characteristics and tendencies to respond to certain situations, acquired in a single assessment. State assessment could be equally valuable but better served by very dense experience sampling of answers to a small set of tailored brief statements in ecological momentary assessment protocols, e.g.^13^ This would require a different approach, focused on capturing contexts, triggers and responses. The procedure resulted in selection of 101 of the 221 items to be included in the exploratory factor analysis.

#### Exploratory factor analysis

To evaluate the dimensionality of hyperarousal, we performed EFA with a principal factor solution and promax rotation on the polychoric correlation matrix of the 101 selected hyperarousal items. Promax was preferred over oblimin because it less computationally intensive and is recommended for larger factor models. The number of factors was determined using parallel analysis (Figure S1) and construct validity. In line with recommendations, items with a loading ≥ |0.32| were considered representative for a factor (Table S7).^27^ Validity of the factors was investigated by inspecting the content of the high loading items. To obtain scores for each factor for each participant, their responses were multiplied by the corresponding item loadings.

#### Hyperarousal dimensions

To obtain a comprehensive yet concise set of items representing the observed different hyperarousal dimensions, we selected 2–5 items for each factor based on high loadings and adequate representation of factor content. Five factors are represented by 4 items each. Somatic hyperarousal is represented by 5 items to cover a wide range of somatic experiences. Vigilant hyperarousal is represented by 2 items due to limited available items and cross-loadings. To evaluate the factor structure, we performed a second EFA on the 27 selected items and performed a within-sample confirmatory factor analysis using the ‘lavaan’ package. To obtain weighted factor scores based on the selected items we rescaled the item responses to a common range (0–4) and calculated the mean value across the selected items within each hyperarousal factor. As a result, hyperarousal dimension factor scores can theoretically range from 0 to 4 with higher values indicating higher levels of experienced hyperarousal in that dimension. Due to technical problems in the first assessment round, 44 participants had 1 or 2 hyperarousal items with missing value(s). As a result their 1 or 2 associated hyperarousal dimension scores were missing. Subsequent analyses were performed using pairwise complete data.

#### Representation of hyperarousal dimensions in different diagnoses

Multiple regression models were used to reveal profiles of the most relevant hyperarousal dimensions associated with the symptom severity of ID, MDD, GAD, SAD, PD, PTSD and ADHD. Age and sex were included as covariates. Standardized regression coefficients were obtained by scaling continuous variables prior to fitting the regression models. Confidence intervals were estimated based on t-values using ‘confint’ from the ‘stats’ package. Complementary to the multiple linear regression models, we used the ‘bootnet’ package^28^ to estimate the network of partial correlations between mental disorder severities and hyperarousal dimension scores. In the estimation, we used the EBICglasso that employs LASSO regularization^29^ to reduce the number of spurious edges and aims to select a model that generalizes well to new data. We used the Extended Bayesian Information Criterion (EBIC) with γ = 0.5 to determine the optimal amount of regularization that was applied.^30^ Edge weight stability was estimated using non-parametric bootstrap (2000 samples) and edges with a significance level, based on bootstrapped confidence intervals, below 0.05 were set to zero using ‘bootThreshold’.

#### Transdiagnostic hyperarousal dimensions questionnaire

To obtain a practical self-report tool, we used the 27 selected items representing the seven dimensions of hyperarousal to create the THDQ. We created an introductory instruction, rephrased items into statements, included item specific instructions for grouped statements and used a uniform 5-point Likert-type response scale with the answer options not, slightly, moderately, considerably and, strongly, respectively scored from 0 to 4. The THDQ was developed in Dutch and translated in English and German using back-and-forth translation. The three versions of the THDQ are included in the supplemental materials. To assess robustness and evaluate whether initial fit indices from the within-sample CFA were the result of overfitting, we performed a confirmatory factor analysis on the THDQ data obtained in the second assessment using the ‘lavaan’ package and in addition estimated Chronbach's α and Mcdonald's ω using the ‘psych’ package. Combining the data of the 315 people who participated in both assessments allowed us to calculate Pearson correlations to evaluate the consistency of their THDQ dimension scores with the dimension scores based on the full item set of the first assessment.

### Sensitivity analyses

To assess the association between hyperarousal dimension scores and key demographic variables we calculated Pearson correlations for age and years of education, and Spearman correlations for socioeconomic status (income categories were numbered from low to high with “prefer not to say” treated as missing). Finally, we repeated the multiple regression analyses with an extended set of covariates including age, sex, socioeconomic status and years of education.

#### Hyperarousal in the UK Biobank

To examine the possibility to estimate hyperarousal dimension factor scores using available UKB items, we calculated the polychoric correlation between responses on the 27 selected items and responses on the 22 UK Biobank items obtained in the first assessment. To obtain the loadings of each UKB item on each of the hyperarousal factors in the original EFA, we applied Dwyer's factor extension^31^ using fa. extension from the R-package ‘psych’. Our aim was to select specific and robust UK Biobank items to estimate the hyperarousal dimension scores. In line with convention, we therefore decided to select UK Biobank items with high loading (>|0.45|, corresponding to ∼ 20% explained variance) on one factor and not having any cross loadings (>|0.32|, corresponding to ∼ 10% explained variance) on any other factor.^27^ Response “prefer not to answer” and “Do not know” were treated as missing and excluded. The remaining responses were rescaled to a common range (0–4). UKB-based hyperarousal dimension score estimates were calculated by taking the mean across the items within a factor. Finally, Pearson correlations were calculated to evaluate correspondence between the hyperarousal dimension factor scores and the UK Biobank estimates of these scores.

### Role of the funding source

The funders had no role in the design, data collection, data analysis, and reporting of this study.

## Results

### Participants

All questionnaires required for analyses were completed by 467 participants in the first assessment (see Table 1 and Table S2), mostly female (77.6%), with a mean age of 58.3 (standard deviation 13.8, range 21–89 years). Most participants had Dutch origins (86.0%) and a large proportion (74.9%) had completed at least higher vocational education. As intended and expected for our specifically selected sample, mental health symptoms of varying nature and severity were common (see Tables S1–S2). Based on cut-off scores on the RMT20, ISI and ASRS, 135 participants were unlikely to have any mental disorder while 332 participants fulfilled the criteria for one (n = 138) or multiple (n = 194) mental disorder diagnoses (see Table S2).

**Table 1:** Sample characteristics.

	All
n	467
Age^a^^,^^c^	58.26 (13.79)
Female^b^^,^^c^	356 (77.6)
Years of education^a^^,^^c^	10.71 (3.58)
Symptom severity	
ID^a^	12.99 (7.04)
MDD^a^	8.27 (4.12)
GAD^a^	9.78 (4.26)
SAD^a^	8.17 (4.07)
PD^a^	5.31 (2.49)
PTSD^a^	6.48 (4.16)
ADHD^a^	2.26 (1.77)

ID, insomnia disorder; MDD, major depressive disorder; GAD, generalized anxiety disorder; SAD, social anxiety disorder; PD, panic disorder; PTSD, posttraumatic stress disorder; ADHD, attention deficit/hyperactivity disorders.

a Mean (SD).

b n (%).

c n = 459.

### Exploratory factor analysis

We performed EFA to identify hyperarousal dimensions. The available 221 items were reduced to 101 to prevent excessive multicollinearity and obtain a meaningful factor structure (see Methods). Parallel analysis initially suggested 16 factors, but based on inspection of the plot (Figure S1) and factor content, we continued with 7 factors which explained 50.2% of the variance in the data. Model solutions with more than 7 factors showed reduced content validity and limited gain in explained variance. Table S7 provides an overview of the included items and their factor loadings. The factors represented different hyperarousal domains and accounted for 32%, 19%, 14%, 12%, 10%, 7% and 6% of explained variance. Model-derived correlations between factors were moderate (median 0.40, range 0.14–0.57).

When examining item loadings ≥ |0.32|, the first factor consisted of 36 items (loading = −0.33 to 0.82) related to future adversity and worry, and was labelled as “anxious hyperarousal”. The second factor consisted of 22 items (loading = 0.35–0.88) representing somatic symptoms and was labelled as “somatic hyperarousal”. The third factor consisted of 20 items (loading = −0.34 to 0.65) representing emotional vulnerability and sensitivity and therefore labelled as “sensitive hyperarousal”. The fourth factor consisted of 11 items (loading = 0.34–0.84) representing pre-sleep rumination and sleep difficulties, therefore labelled as “sleep-related hyperarousal”. The fifth factor consisted of 7 items (loading = 0.47–0.83) representing irritability and was labelled as “irritable hyperarousal”. The sixth factor consisted of 6 items (loading = 0.36–0.74) related to vigilance and awareness in public spaces, therefore labelled as “vigilant hyperarousal”. Finally, the seventh factor consisted of 5 items (loading = 0.46–0.69) on hot or cold sweats and facial flushing, therefore labelled as “sudomotor hyperarousal”.

### Dimensions of hyperarousal

**Fig. 1:**
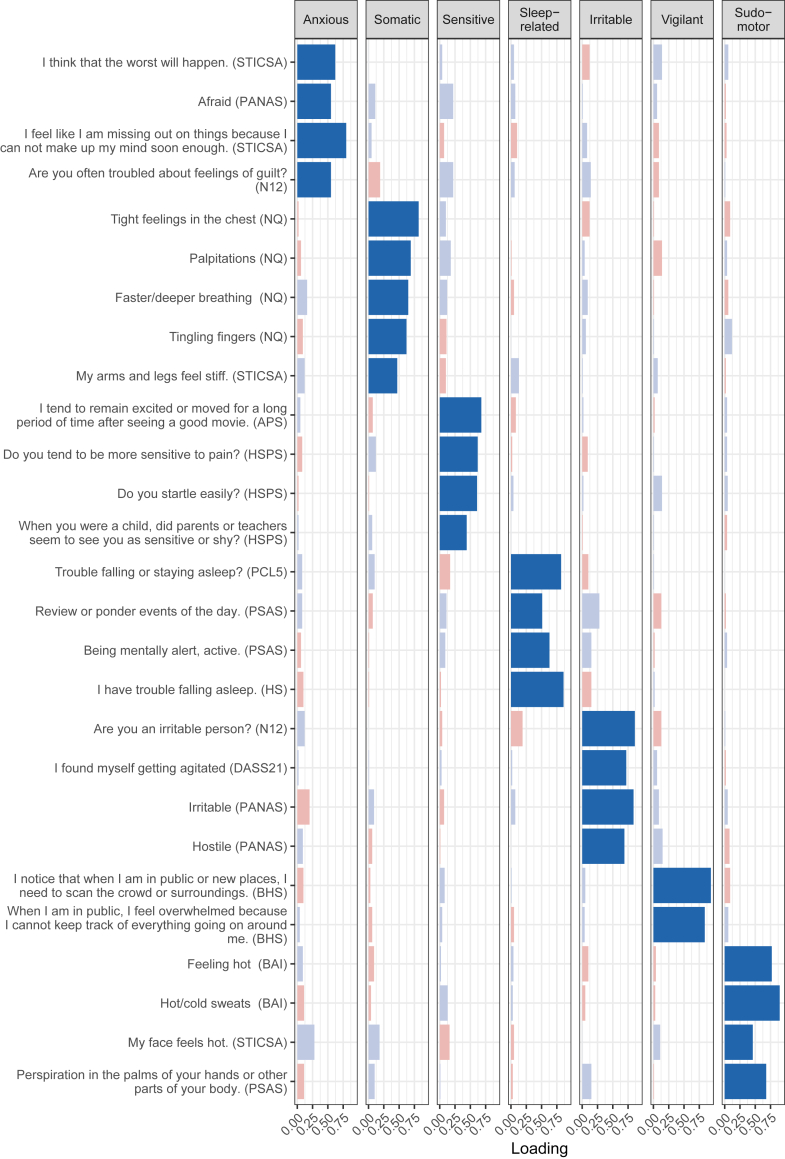
Factor loadings from the exploratory factor analysis of the 27 selected items reflecting the dimensions of hyperarousal. Positive factor loadings are shown in blue and negative factor loadings in red. Color opacity reflects loading strength. STICSA, State-Trait Inventory for Cognitive and Somatic Anxiety; PANAS, Positive and Negative Affect Schedule; N12, Eysenck Neuroticism 12; NQ, Nijmegen Hyperventilation Questionnaire; APS, Arousal predisposition scale; HSPS, High Sensitive Person Scale; PCL5, PTSD checklist for dsm-5; PSAS, Pre-sleep arousal scale; HS, Hyperarousal scale; DASS21, Depression, Anxiety and Stress Scale; BAI, Beck Anxiety Inventory.

To attain a concise questionnaire for the complete assessment the seven identified hyperarousal dimensions, 2–5 items were selected for each factor based on high loadings and adequate representation of factor content, resulting in 27 items. EFA on the 27 selected items confirmed the initial 7-factor structure (see Fig. 1) and confirmatory factor analysis within the first sample indicated an acceptable fit range (comparative fit index [CFI] = 0.923, Tucker Lewis Index [TLI] = 0.911, root mean square error of approximation [RMSEA] = 0.048, standardized root mean square residual [SRMR] = 0.050). Trying to enforce a reduced 6-factor model combining the two factors with somatic symptoms (somatic hyperarousal and sudomotor hyperarousal, r_scores_ = 0.67) resulted in a significantly poorer fit (p = 2.2 × 10^−16^, CFI = 0.883, TLI = 0.867, RMSEA = 0.058, SRMR = 0.055). While our aim was to explore the factor structure of hyperarousal-related items, we additionally explored an higher-order model in which all dimensions load onto a general hyperarousal factor. Compared to the original 7-factor model, the higher-order model showed a similar, but significantly poorer fit (p = 5.8 × 10^−7^, CFI = 0.912, TLI = 903, RMSEA = 0.050, SRMR = 0.057). Conducting EFA and CFA within the same sample comes with limitations, we therefore conducted a second CFA on new data from the concise questionnaire (see *Transdiagnostic hyperarousal dimensions questionnaire*). Hyperarousal factor scores based on the 27 selected items were highly correlated with the corresponding factor scores based on all 101 items: r_anxious_ = 0.86, r_somatic_ = 0.92, r_sensitive_ = 0.87, r_sleep_-_related_ = 0.91, r_irritable_ = 0.89, r_vigilant_ = 0.82, r_sudomotor_ = 0.90. The correlation between factors was lower for the hyperarousal factor scores based on the 27 selected items (r = 0.24–0.49) as compared to the factor scores based on all 101 items (r = 0.33–0.76). Correlations between hyperarousal dimension scores and scores on the existing hyperarousal (sub)scales, showed that the latter differentially represented mixtures of the seven dimensions (see Figure S2).

### Representation of hyperarousal dimensions in different mental disorders

The participants (n = 135) that were unlikely to have any mental disorder diagnosis had low scores on all seven hyperarousal dimensions, while still showing non-zero variance. Participants with clinically relevant symptoms of at least one mental disorder had significant higher scores on all dimensions (see Figure S3). Multiple regression analyses evaluating the contribution of each hyperarousal dimension to the severity of each disorder revealed that different hyperarousal dimensions rank highest in association with different symptom type severities (see Table 2 for an overview of the significant coefficients and Table S8 for all coefficients including confidence intervals): Anxious hyperarousal with GAD, Somatic hyperarousal with PD, Sensitive hyperarousal with SAD, Sleep-related hyperarousal with ID, Irritable hyperarousal with MDD, Vigilant hyperarousal and Sudomotor hyperarousal with PTSD. None of the seven hyperarousal dimensions ranked highest for the association with ADHD symptom severity. This does not mean that hyperarousal dimensions do not contribute to ADHD symptom severity. For example, irritable hyperarousal contributed more to the severity ADHD symptoms than of all other disorders except MDD. Not every dimension of hyperarousal contributed significantly to each of the symptom severities. Sensitive hyperarousal was a significant predictor only for the severity of symptoms of MDD (β = −0.10, p = 0.01) and SAD (β = 0.18, p = 2.37 × 10^−5^. Sudomotor hyperarousal only contributes little to the symptom severity of each of the disorders (Table S8), reaching significance only for PTSD (β = 0.12, p = 4.98 × 10^−3^). Comparing hyperarousal dimensions, anxious hyperarousal correlated most strongly across MDD, GAD, SAD, PD, PTSD and ADHD severities symptom. Regression models with only one dimension revealed that all hyperarousal dimensions were significantly associated with the severity of every disorder (Table S9).

**Table 2:** Contribution of each hyperarousal dimension to the severity of symptoms characterizing each disorder.

	ID	MDD	GAD	SAD	PD	PTSD	ADHD
Anxious	–	0.43 (0.04)	**0.51 (0.04)**	0.32 (0.05)	0.37 (0.05)	0.31 (0.05)	0.25 (0.05)
Somatic	0.08 (0.04)	0.10 (0.04)	0.12 (0.04)	0.09 (0.04)	**0.22 (0.05)**	0.10 (0.05)	–
Sensitive	–	−0.10 (0.04)	–	**0.18 (0.04)**	–	–	–
Sleep-related	**0.70 (0.04)**	0.20 (0.04)	0.23 (0.03)	0.11 (0.04)	–	–	0.14 (0.05)
Irritable	0.09 (0.04)	**0.19 (0.04)**	0.10 (0.03)	0.10 (0.04)	–	–	0.14 (0.05)
Vigilant	–	–	–	0.20 (0.04)	0.19 (0.04)	**0.25 (0.04)**	–
Sudomotor	–	–	–	–	–	**0.12 (0.04)**	–

Significant standardized beta-coefficients (standard error) from the multiple regression analyses evaluating the contribution of each hyperarousal dimension to the severity of symptoms characterizing each disorder. Note that different hyperarousal dimensions show the largest effect size (in bold) in association with different severities of symptoms characterizing each disorder (left-right): Anxious with GAD, Somatic with PD, Sensitive with SAD, Sleep with ID, Irritable with MDD, Vigilant and Sudomotor with PTSD. ID, insomnia disorder; MDD, major depressive disorder; GAD, generalized anxiety disorder; SAD, social anxiety disorder; PD, panic disorder; PTSD, posttraumatic stress disorder; ADHD, attention deficit/hyperactivity disorders.

**Fig. 2:**
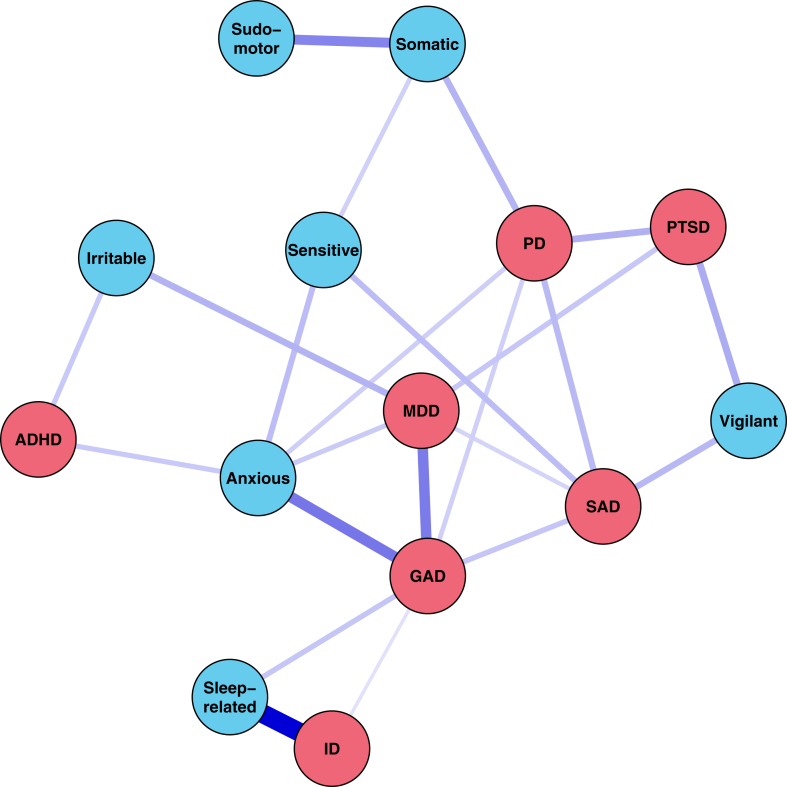
Network graph of the partial correlations between severities of the different hyperarousal dimensions and mental disorder symptoms. Opacity and thickness of edges indicate connection strength. ID, insomnia disorder; MDD, major depressive disorder; GAD, generalized anxiety disorder; SAD, social anxiety disorder; PD, panic disorder; PTSD, posttraumatic stress disorder; ADHD, attention deficit/hyperactivity disorders.

Complementary to the seven linear models, we calculated a partial correlation network between all hyperarousal dimension scores and mental disorder severities, where each association reflects the unique relation between two variables after adjusting for all others (see Fig. 2) allowing us to assess indirect connections between hyperarousal dimensions and mental disorders. After bootstrapping and thresholding, we observed a sparse network with 23 out of the 91 possible connections (25%) (see Figure S4 for edge stability as a measure of fit and Table S10 for edge weights). Largely overlapping with the results of the multiple regression analysis, the network graph suggests direct associations of: Anxious hyperarousal with GAD but also with MDD, ADHD and PD; Somatic hyperarousal with PD, Sensitive hyperarousal with SAD, Sleep-related hyperarousal with ID but also with GAD; Irritable hyperarousal with MDD but also with ADHD, Vigilant hyperarousal with PTSD but also with SAD, while Sudomotor hyperarousal connected to PTSD only indirectly through its association with PD and Somatic hyperarousal. Indirect connections, i.e. Somatic—PD—PTSD, suggest that associations between hyperarousal dimensions and the severity of different mental disorders may be mediated by another dimension or by symptoms of another mental disorders.

### Transdiagnostic hyperarousal dimensions questionnaire

A concise THDQ was created by slightly rephrasing selected items and instructions to arrive at a coherent questionnaire with a consistent format and uniform response options (see supplement). Confirmatory factor analysis of the THDQ in a second sample of 592 people (see Table S11 , 65.2% female, mean age = 61.0) indicated an acceptable fit range (CFI = 0.919, TLI = 0.906, RMSEA = 0.054, SRMR = 0.051, Chronbach's α = 0.90, Mcdonald's ω total = 0.93 and ω hierarchical = 0.73). Chronbach's α values for individual dimensions were 0.75 for anxious, 0.77 for somatic, 0.65 for sensitive, 0.81 for sleep-related, 0.86 for irritable, 0.82 for hypervigilance and 0.68 for sudomotor hyperarousal. Formal test-retest analysis was not possible due to the required rephrasing to obtain a consistent and uniform THDQ. However, to evaluate within-sample consistency over time we analyzed a subset of 315 people (75.6% female, mean age = 60.8) who filled out the THDQ on average 59.9 ± 5.9 weeks after they had completed the original extensive set of questions. Factor scores based on the 27 selected items and THDQ dimension scores showed moderate to good Pearson correlations (r_anxious_ = 0.77, r_somatic_ = 0.64, r_sensitive_ = 0.81, r_sleep-related_ = 0.77, r_irritable_ = 0.69, r_vigilant_ = 0.60, r_sudomotor_ = 0.63).

### Sensitivity analyses

To assess robustness of the hyperarousal dimension scores we examined correlations with age, years of education and social economic status. Additional analyses for race were not conducted due to limited non-Dutch participants (see Table S2). Hyperarousal dimension scores based on the 27 selected items in the first sample showed low correlations with age (r = −0.23 to −0.11), years of education (r = −0.13 to −0.04) and socioeconomic status (rho = −0.17 to −0.08). Hyperarousal dimensions scores from the THDQ in sample two showed similar low correlations with age (r = −0.29 to −0.06), years of education (r = −0.13 to 0.06) and socioeconomic status (rho = −0.15 to 0.02). Finally, to evaluate the sensitivity of multiple regression analyses we repeated them with additional covariates beyond age and sex. The inclusion of socioeconomic status and years of education did not significantly alter the results (see Table S12).

### Hyperarousal in the UK Biobank

With over 500.000 participants, a mean age of 56.5 years at recruitment, 54.4% female and 46.0% with a college or university degree, the UK Biobank is a large cohort that aligns with our samples (see Table S13).^32^ While the UK Biobank does not include a dedicated hyperarousal questionnaire, individual items in included questionnaires might be representative for one or more of the seven hyperarousal dimensions. Using the polychoric correlations between the 27 selected items and the selected relevant UK Biobank items included in our assessment, allowed us to estimate their loadings on each of the hyperarousal dimensions. UK Biobank items with a high factor loading of >|0.45| on one of the hyperarousal dimensions, while not having any cross loadings >|0.32| on one of the other dimensions were selected to obtain specific and robust estimates for the seven hyperarousal dimensions (see Table S14). Three hyperarousal dimensions were sufficiently represented by available UK Biobank items, with eight items for anxious hyperarousal, five items for irritable hyperarousal and four items for sleep-related hyperarousal. The UK Biobank-derived estimates for these three hyperarousal dimensions correlated strongly with the TDHQ scores on these dimensions (r_anxious_ = 0.83, r_irritable_ = 0.85, r_sleep-related_ = 0.75).

## Discussion

Our study aimed to open up transdiagnostic study of hyperarousal, considered to be a key symptom of multiple mental disorders including insomnia, anxiety and stress disorders. To do so, we first performed a comprehensive assessment in a large sample of people with a wide variety of symptoms of these mental health conditions. Among the commonly used disorder-specific hyperarousal questionnaires, we identified seven dimensions. The dimensions represent anxious-, somatic-, sensitive-, sleep-related-, irritable-, vigilant- and sudomotor hyperarousal experiences. All seven hyperarousal dimension scores were low in people without any likely mental health diagnosis, while consistently elevated in people with clinically relevant severity of symptoms for at least one mental health condition. Moreover, different profiles of increased hyperarousal dimensions were associated with the severity of symptoms of ID, MDD, GAD, SAD, PD, PTSD and ADHD. This suggests that while hyperarousal dimensions are transdiagnostic, the profile of their severities has some disorder-specificity. The finding moreover supports the hypothesis that hyperarousal is key to the severity of many mental disorders.

The seven hyperarousal dimensions distinguish multiple cognitive, emotional, and somatic features of the tension and distress experienced as hyperarousal. Sensitive hyperarousal was an interesting finding and likely represents a more reactive dimension with items related to heightened and prolonged responsiveness to stimuli. While related to anxious hyperarousal in the network analyses, sensitive hyperarousal conceptually differs from the general fear and worry associated with anxious hyperarousal. We showed that all the existing, usually disorder-specific, questionnaires reflect different mixtures of the seven dimensions. Moving towards studying transdiagnostic hyperarousal provides an interesting opportunity to asses if the brain correlates of hyperarousal dimensions can improve reliability and reproducibility, a common challenge when studying brain structure and function related to mental disorders.^33^, ^34^, ^35^, ^36^, ^37^ Furthermore, more precise differentiation of hyperarousal may improve our understanding of treatment responses, expanding and building on previous research showing that pre-sleep arousal mediates the effect of cognitive behavioural therapy for insomnia on disorder severity.^38^ Similarly, the novel dimensions of hyperarousal may refine the link between hyperarousal and inflammation suggested in PTSD.^39^^,^^40^ Distinguishing actual transdiagnostic hyperarousal dimensions may better capture differential relevance to disease severity, prognosis and treatment response, as well as facilitate understanding of underlying mechanisms including genetic risk, early life stress, and brain structure and function.

To facilitate future transdiagnostic research on hyperarousal, we developed the 27-item THDQ which is available in Dutch, English and German (see supplement). The THDQ allows scholars and clinicians targeting different mental disorders to uniformly assess the different dimensions of hyperarousal in a standardized manner. While formal test-retest analysis was not possible in the current datasets, confirmatory factor analysis showed an adequate fit in a second large sample and consistency over time, indicated by moderate to high correlations between original hyperarousal dimension scores and THDQ scores assessed more than a year later. While extensive measurement invariance testing is required to make sure that this new scale functions similarly across relevant subgroups (e.g. sex, age), such analyses could at present not be conducted as the complexity of the model requires larger subgroup samples. Future research should further validate test-retest reliability of the 27-item THDQ assessed twice and asses measurement invariance of the THDQ across relevant groups. The transdiagnostic value relative to existing hyperarousal questionnaires is that this single questionnaire can be used across disorders because it covers hyperarousal in multiple dimensions. The profile of hyperarousal severity contributes to distinguishing disorders or recognizing comorbidity or underlying other pathology in individual patients. For example, in a patient presenting with insomnia, a profile where high sleep-related hyperarousal is accompanied by relatively high scores on the Anxious, Vigilant and Sudomotor dimensions may point the clinician to possible underlying posttraumatic stress pathology.

Lastly, we demonstrated that available UK Biobank items allow to approximate anxious-, irritable- and sleep-related hyperarousal scores. While the UK Biobank lacks a dedicated hyperarousal questionnaire, we showed that it is possible to reliably estimate the severity of symptoms in these three hyperarousal dimensions using available data. The possibility to estimate these three hyperarousal dimensions opens fast avenues for transdiagnostic studies on risks as well as genetic and brain structural and functional correlates of well-defined hyperarousal dimensions in a large dataset. It can be challenging to find robust biological, physiological and behavioural correlates of psychiatric symptoms, which are largely assessed from subjective reporting. The THDQ may provide a small step toward bridging, by reducing unexplained heterogeneity. For example, assessing profiles of dimensions rather than single severity scores has facilitated finding brain structural and functional correlates of insomnia.^41^^,^^42^

A first example of how scores on the three hyperarousal dimensions in the UK Biobank sample may be used would be to study risk factors leading to hyperarousal and risks conveyed by hyperarousal. For example previous smaller studies have suggested that adverse childhood experiences (ACE) predict the hyperarousal severity in PTSD^43^^,^^44^ and that hyperarousal severity in turn mediates effects of ACE on physical health.^43^ The three hyperarousal dimension estimates combined with the adverse life events measures in UKB participants may be used to more precisely delineate the association between life events, hyperarousal and mental health.

The UK Biobank also offers the opportunity to study brain structural and functional correlates of the three hyperarousal dimension severity estimates. Previous neuroimaging studies on hyperarousal are primarily focused on disorder-specific brain-correlates, nevertheless providing valuable insights. For example, studies on insomnia suggested involvement of the dorsal anterior cingulate cortex, part of the limbic and salience circuitry in hyperarousal.^45^^,^^46^ In a study on anxiety, subjective anxious arousal measured with the uncertainty-variation threat anticipation paradigm was associated with a distributed neural representation of brain activity including multiple brain regions such as the thalamus, putamen, anterior insula and anterior cingulate cortex.^47^ The possibility to reliably assess three distinct hyperarousal dimensions within the available UK Biobank data will allow for a better delineation of brain circuits associated with these three hyperarousal dimensions. Finally, genome-wide association studies in the UK Biobank sample may be used to study the genetic predisposition each of three hyperarousal dimensions, and thus contribute to a better understanding of the shared biological processes underlying the observed genetic correlations between hyperarousal-related mental disorders like insomnia, MDD, anxiety and ADHD.^19^^,^^48^

Some limitations can be mentioned. First, the current approach is strictly based on subjective experiences. Future studies may include central and autonomic indicators of hyperarousal, as well as evaluate how they map on the seven dimensions. Second, we studied a home-dwelling sample using three questionnaires to assess symptom severity for seven mental disorders. While the recruitment, primarily through the NSR, was such that we successfully included both people unlikely to have any mental disorder and many people with probable clinically relevant symptom severity of several mental disorders, there remains a risk of overrepresentation e.g. of people experiencing sleep problems. It would therefore be useful to further evaluate the hyperarousal dimensions in future epidemiological studies. From a clinical perspective, it would be of interest to study clinical samples of hospitalized and outpatient participants using more extensive clinical assessment. Third, our samples had a relative older mean age and higher educational attainment which could impact generalizability to younger people and or people with lower educational attainment. While hyperarousal dimension scores showed low correlations with age and educational attainment, future studies should validate generalizability in these populations. Fourth, our current sample included more females than males. In the age range of the current sample, this imbalance matches the imbalance in the risk of many psychiatric disorders.^49^ However, the imbalance is mostly driven by the overall more prevalent internalizing disorders for which women are at increased risk, while the less prevalent externalizing disorders are more prevalent in males. We therefore cannot exclude the possibility of additional types of hyperarousal that occur mostly in males and externalizing disorders.

In conclusion, the current study revealed seven hyperarousal dimensions that are differentially associated with the severity of symptoms of insomnia-, major depression, anxiety-, posttraumatic stress and attention deficit hyperactivity disorders. The compiled new THDQ allows researchers and clinicians to validly assess seven distinct hyperarousal dimensions with a concise questionnaire. We moreover facilitated the study of three hyperarousal dimensions in the large population cohort of the UK Biobank data. Distinguishing different hyperarousal dimensions can propel our understanding of the mechanisms underlying the key role of various types of hyperarousal in multiple mental disorders, and lead to better assessment and treatment.

## Contributors

**Tom Bresser**: Writing original draft, Writing review & editing, Formal analysis, Data curation, Conceptualization, Accessed and verified the data. **Siemon de Lange**: Writing review & editing, Formal analysis, Data curation, Conceptualization. **Lara Rösler**: Writing review & editing, consultation statistical analyses. **Tessa F. Blanken**: Writing review & editing, consultation network analyses. **Sophie van der Sluis**: Writing review & editing, consultation statistical analyses. **Eus JW. van Someren**: Writing—review & editing, Supervision, Data curation, Conceptualization, Accessed and verified the data.

## Data sharing statement

The data supporting the findings of this study are not publicly available as public data sharing for any purpose was not explicitly part of informed consent. The data are available from the corresponding author [T.B.] or last author [E.S.] upon reasonable request.

## Declaration of interests

SvdS is supported by NWO Gravitation: BRAINSCAPES: A Road map from Neurogenetics to Neurobiology (Grant No. 024.004.012). TFB is supported by NWO Veni Grant (nr 231G.083) and has received honorarium for lecture in Comenius Leadership program. All other authors declare no competing interests.

## Appendix A Supplementary data

Supplementary Figures and Tables

Translated Abstract
