# Supplementary material for: Hyperarousal transdiagnostically dissected: different dimensions characterize mood, anxiety, insomnia, posttraumatic stress and attention deficit hyperactivity disorders
Source: eClinicalMedicine. 2026 Mar 12;94:103810. doi: 10.1016/j.eclinm.2026.103810 (PMC13133538; doi:10.1016/j.eclinm.2026.103810)
Supplement: Supplementary Figures and Tables [file mmc1.docx]

**Supplementary material**

Hyperarousal transdiagnostically dissected: Different dimensions characterize mood, anxiety, insomnia, posttraumatic stress and attention deficit hyperactivity disorders

**Table s1** p2-3

**Table s2** p4-5

**Table s3** p5

**Table s4** p6

**Table s5** p7

**Table s6** p7

**Figure s1** p8

**Table s7** p9-12

**Figure s2** p13

**Figure s3** p14

**Table s8** p15

**Table s9** p16

**Table s10** p16

**Figure s4** p17-18

**Table s11** p18

**Table s12** p19

**Table s13** p20

**Table s14** p20

**Transdiagnostic hyperarousal dimensions questionnaire (NL)** p21-22

**Transdiagnostic hyperarousal dimensions questionnaire (EN)** p23-24

**Transdiagnostic hyperarousal dimensions questionnaire (DU)** p25-26

**Table s1.** Overview of self-reported lifetime mental health or sleep disorder diagnoses.

|  | **Self-diagnose** | |  | **Confirmed by a professional** | |
| --- | --- | --- | --- | --- | --- |
|  | **Currently** | **In the past** |  | **Currently** | **In the past** |
| **Any psychiatric disorder** | 162 (34.7%) | 267 (57.2%) |  | 102 (21.8%) | 183 (39.2%) |
| **Depression** | 59 (12.6%) | 164 (35.1%) |  | 41 (8.8%) | 121 (25.9%) |
| **Mania-hypomania-bipolar or manic depressive** | 4 (0.9%) | 13 (2.8%) |  | 3 (0.6%) | 9 (1.9%) |
| **Schizophrenia** | 1 (0.2%) | 0 (0.0%) |  | 0 (0.0%) | 0 (0.0%) |
| **Any other type of psychosis or psychotic disorder** | 5 (1.1%) | 15 (3.2%) |  | 4 (0.9%) | 13 (2.8%) |
| **Personality disorder** | 21 (4.5%) | 35 (7.5%) |  | 17 (3.6%) | 33 (7.1%) |
| **Autism-asperger or autistic spectrum disorder** | 25 (5.4%) | 25 (5.4%) |  | 16 (3.4%) | 18 (3.9%) |
| **Attention deficit or attention deficit hyperactivity disorder ADD ADHD** | 32 (6.9%) | 35 (7.5%) |  | 16 (3.4%) | 19 (4.1%) |
| **Obsessive compulsive disorder OCD** | 15 (3.2%) | 15 (3.2%) |  | 6 (1.3%) | 8 (1.7%) |
| **Anxiety disorder** | 49 (10.5%) | 83 (17.8%) |  | 32 (6.9%) | 61 (13.1%) |
| **Generalized anxiety disorder** | 32 (6.9%) | 52 (11.1%) |  | 24 (5.1%) | 41 (8.8%) |
| **Social anxiety or social phobia** | 29 (6.2%) | 60 (12.8%) |  | 10 (2.1%) | 21 (4.5%) |
| **Agoraphobia** | 6 (1.3%) | 11 (2.4%) |  | 4 (0.9%) | 6 (1.3%) |
| **Any other phobia e.g. paralyzing fear of heights or spiders** | 38 (8.1%) | 48 (10.3%) |  | 5 (1.1%) | 13 (2.8%) |
| **Panic attacks** | 27 (5.8%) | 85 (18.2%) |  | 17 (3.6%) | 32 (6.9%) |
| **Panic disorder** | 16 (3.4%) | 24 (5.1%) |  | 10 (2.1%) | 15 (3.2%) |
| **Posttraumatic stress disorder PTSD** | 29 (6.2%) | 66 (14.1%) |  | 21 (4.5%) | 51 (10.9%) |
| **Anorexia nervosa** | 2 (0.4%) | 14 (3.0%) |  | 2 (0.4%) | 8 (1.7%) |
| **Bulimia nervosa** | 1 (0.2%) | 16 (3.4%) |  | 0 (0.0%) | 7 (1.5%) |
| **Binge eating disorder** | 6 (1.3%) | 11 (2.4%) |  | 0 (0.0%) | 2 (0.4%) |
| **Any other eating disorder** | 12 (2.6%) | 20 (4.3%) |  | 3 (0.6%) | 7 (1.5%) |
| **Any sleep disorder** | 191 (40.9%) | 226 (48.4%) |  | 87 (18.6%) | 119 (25.5%) |
| **Insomnia** | 120 (25.7%) | 134 (28.7%) |  | 53 (11.3%) | 74 (15.8%) |
| **Hypersomnia** | 2 (0.4%) | 8 (1.7%) |  | 0 (0.0%) | 1 (0.2%) |
| **Circadian rhythm disorder** | 12 (2.6%) | 15 (3.2%) |  | 5 (1.1%) | 8 (1.7%) |
| **Parasomnia or sleep related problem e.g. sleepwalking bedwetting teeth grinding** | 39 (8.4%) | 70 (15.0%) |  | 17 (3.6%) | 27 (5.8%) |
| **Restless legs RLS** | 70 (15.0%) | 82 (17.6%) |  | 17 (3.6%) | 23 (4.9%) |
| **Periodic leg movements in sleep PLMS** | 29 (6.2%) | 33 (7.1%) |  | 6 (1.3%) | 13 (2.8%) |
| **Sleep apnea** | 28 (6.0%) | 35 (7.5%) |  | 23 (4.9%) | 28 (6.0%) |

*Self-report of (mental) health problems that participants currently experience or have experienced in the past. Using checkboxes participants reported a current and/or past mental health diagnosis and if the diagnosis was made by a professional (doctor, nurse or another specialist).*

**Table s2.** Demographics of sample 1.

|  | **All** | **ID** | **MDD** | **GAD** | **SAD** | **Panic** | **PTSD** | **ADHD** |
| --- | --- | --- | --- | --- | --- | --- | --- | --- |
| **n** | 467 | 295 | 62 | 165 | 68 | 36 | 103 | 63 |
| **Age ^1,3^** | 58.26 (13.79) | 57.65 (13.51) | 51.51 (14.55) | 55.02 (14.29) | 55.86 (13.12) | 51.09 (15.33) | 53.21 (13.75) | 52.19 (14.14) |
| **Female ^2,3^** | 356  (77.6) | 230  (79.0) | 46  (78.0) | 133  (82.6) | 46  (68.7) | 29  (82.9) | 83  (81.4) | 48  (77.4) |
| **Years of education ^1,3^** | 10.71 (3.58) | 10.51 (3.70) | 10.10 (3.67) | 10.39 (3.72) | 10.33 (3.88) | 10.60 (3.82) | 10.42 (3.68) | 10.48 (3.80) |
| **Symptom severity** |  |  |  |  |  |  |  |  |
| **ISI ^1^** | 12.99 (7.04) | 17.44 (4.35) | 19.58 (5.35) | 17.18 (6.08) | 17.62 (6.05) | 18.81 (6.20) | 17.11 (6.48) | 18.11 (6.07) |
| **RMT20 MDD ^1^** | 8.27 (4.12) | 9.41  (4.30) | 15.84 (1.73) | 11.81 (3.68) | 12.44 (3.52) | 13.17 (3.25) | 12.16 (3.83) | 11.54 (4.62) |
| **RMT20 GAD ^1^** | 9.78 (4.26) | 11.17 (4.24) | 15.24 (3.23) | 14.66 (2.26) | 14.59 (2.66) | 15.94 (2.60) | 14.05 (3.43) | 13.08 (4.43) |
| **RMT20 SAD ^1^** | 8.17 (4.07) | 9.15  (4.29) | 12.69 (4.47) | 11.38 (4.26) | 15.87 (2.16) | 13.78 (4.45) | 11.83 (4.38) | 11.24 (4.37) |
| **RMT20 panic**  **disorder ^1^** | 5.31 (2.49) | 5.79  (2.87) | 8.18  (3.75) | 7.02  (3.26) | 8.01  (3.81) | 12.28 (2.46) | 7.60  (3.47) | 7.38  (3.76) |
| **RMT20 PTSD ^1^** | 6.48 (4.16) | 7.25  (4.68) | 11.61 (5.63) | 9.41 (5.17) | 10.90 (5.62) | 11.94 (5.91) | 13.56 (3.12) | 10.00 (5.72) |
| **ASRS score ^1^** | 2.26 (1.77) | 2.61  (1.82) | 3.74  (1.72) | 3.19  (1.69) | 3.40  (1.63) | 3.67  (1.90) | 3.42  (1.76) | 5.29  (0.46) |
| **Income ^2,4^** |  |  |  |  |  |  |  |  |
| **< €850** | 10  ( 2.2) | 4  ( 1.4) | 1  ( 1.7) | 3  ( 1.9) | 1  ( 1.5) | 0 ( 0.0) | 3 ( 3.0) | 1 ( 1.6) |
| **€851 - €1150** | 17 ( 3.7) | 12 ( 4.2) | 4 ( 6.8) | 8 ( 5.1) | 4 ( 6.1) | 0 ( 0.0) | 7 ( 6.9) | 3 ( 4.9) |
| **€1151 - €1750** | 46 (10.1) | 35 (12.2) | 11 (18.6) | 22 (13.9) | 11 (16.7) | 5 (14.7) | 20 (19.8) | 10 (16.4) |
| **€1751 - €3050** | 103 (22.6) | 70 (24.4) | 16 (27.1) | 40 (25.3) | 20 (30.3) | 8 (23.5) | 27 (26.7) | 11 (18.0) |
| **€3051 - €3500** | 52 (11.4) | 33 (11.5) | 2 ( 3.4) | 11 ( 7.0) | 4 ( 6.1) | 4 (11.8) | 6 ( 5.9) | 6 ( 9.8) |
| **> €3500** | 155 (34.1) | 85 (29.6) | 16 (27.1) | 46 (29.1) | 20 (30.3) | 14 (41.2) | 26 (25.7) | 20 (32.8) |
| **Prefer not**  **to say** | 72 (15.8) | 48 (16.7) | 9 (15.3) | 28 (17.7) | 6 ( 9.1) | 3 ( 8.8) | 12 (11.9) | 10 (16.4) |
| **Race ^2,5^** |  |  |  |  |  |  |  |  |
| **Dutch** | 394 (86.0) | 248 (85.2) | 46 (78.0) | 136 (84.5) | 56 (83.6) | 29 (82.9) | 83 (81.4) | 55 (88.7) |
| **Mixed** | 42 ( 9.2) | 27 ( 9.3) | 7 (11.9) | 13 ( 8.1) | 4 ( 6.0) | 2 ( 5.7) | 12 (11.8) | 6 ( 9.7) |
| **Turkish,**  **Kurdish** | 1 ( 0.2) | 1 ( 0.3) | 1 ( 1.7) | 1 ( 0.6) | 0 ( 0.0) | 0 ( 0.0) | 1 ( 1.0) | 0 ( 0.0) |
| **European**  **(not Dutch)** | 14 ( 3.1) | 10 ( 3.4) | 3 ( 5.1) | 8 ( 5.0) | 5 ( 7.5) | 4 (11.4) | 5 ( 4.9) | 1 ( 1.6) |
| **Asian** | 5 ( 1.1) | 3 ( 1.0) | 2 ( 3.4) | 2 ( 1.2) | 2 ( 3.0) | 0 ( 0.0) | 1 ( 1.0) | 0 ( 0.0) |
| **African** | 1 ( 0.2) | 1 ( 0.3) | 0 ( 0.0) | 1 ( 0.6) | 0 ( 0.0) | 0 ( 0.0) | 0 ( 0.0) | 0 ( 0.0) |
| **Unknown** | 1 ( 0.2) | 1 ( 0.3) | 0 ( 0.0) | 0 ( 0.0) | 0 ( 0.0) | 0 ( 0.0) | 0 ( 0.0) | 0 ( 0.0) |

*Diagnoses were based on cut-off scores. In total, 332 participants fulfilled the criteria for one
(n=138) or multiple (n=194) mental disorder diagnoses. Race was assessed using self-report on the origin of the biological parents. ISI: Insomnia Severity Index, ID: Insomnia Disorder, MDD: Major depressive Disorder, GAD: Generalized Anxiety Disorder, SAD: Social Anxiety Disorder, Panic: Panic Disorder, PTSD: Post-Traumatic Stress Disorder, ADHD: Attention Deficit Hyperactivity Disorder, ASRS: Adult ADHD Self-Report Scale: ^1^ mean
(SD), ^2^ n (%), ^3^ n = 459, ^4^ n = 455, ^5^ n = 458.*

**Table s3**. Characteristics of participants with incomplete questionnaire data.

|  | | **n** | **Mean (sd) / n (%)** |
| --- | --- | --- | --- |
| **Age ^1,3^** | 266 | | 54.88 (15.39) |
| **Female ^2,3^** | | 266 | 204 (51.0) |
| **Years of education ^1,3^** | | 260 | 10.51 (3.46) |
| **ISI ^1^** | | 305 | 13.90 (7.59) |
| **RMT20 MDD ^1^** | | 305 | 8.97 (4.23) |
| **RMT20 GAD ^1^** | | 305 | 10.80 (4.17) |
| **RMT20 SAD ^1^** | | 305 | 8.86 (4.24) |
| **RMT20 panic disorder ^1^** | | 305 | 5.93 (3.03) |
| **RMT20 PTSD ^1^** | | 305 | 7.03 (4.33) |
| **ASRS score ^1^** | | 305 | 2.69 (1.66) |

*In total 399 participants had incomplete questionnaire data. The table provides an overview of the available demographic data and symptom severity of multiple mental disorders. ISI: Insomnia Severity Index, ID: Insomnia Disorder, MDD: Major depressive Disorder, GAD: Generalized Anxiety Disorder, SAD: Social Anxiety Disorder, Panic: Panic Disorder, PTSD: Post-Traumatic Stress Disorder, ADHD: Attention Deficit Hyperactivity Disorder, ASRS: Adult ADHD Self-Report Scale*

**Table s4.** Hyperarousal-related questionnaires recommended by the consulted expert panel.

|  | **# Items** |
| --- | --- |
| **Insomnia disorder** |  |
| Hyperarousal scale (HS) | 26 |
| Pre-sleep arousal scale (PSAS) | 16 |
| Ford Insomnia Response to Stress Test (FIRST) | 9 |
|  |  |
| **Anxiety disorder** |  |
| PROMIS short – anxiety 8a (PSASF8a) | 8 |
| Beck Anxiety Inventory (BAI) | 21 |
| Hospital Anxiety and Depression Scale (HADS) | 7 |
| State-Trait Inventory for Cognitive and Somatic Anxiety (STICSA): trait | 21 |
|  |  |
| **Post-traumatic stress syndrome** |  |
| Impact of event scale – revised (IES-R): hyperarousal^1^ | 6 |
| PTSD checklist for dsm-5 (PCL-5): hyperarousal^1^ | 6 |
| Brief Hypervigilance Scale (BHS) | 5 |
|  |  |
| **Other** |  |
| Nijmegen Hyperventilation Questionnaire (NQ) | 16 |
| Arousal predisposition scale (APS) | 12 |
| High Sensitive Person Scale (HSPS) | 27 |
| Profile of Mood States (POMS) questionnaire: tension | 6 |
| Depression, Anxiety and Stress Scale (DASS21): stress (STR) | 7 |
| Positive and Negative Affect Schedule (PANAS): negative | 10 |
| NIH toolbox fear questionnaire (FSA): somatic arousal^2^ | 6 |
| Eysenck Neuroticism 12 (N12)^3^ | 12 |
|  | 221 |

*^1^ Modified instructions not requiring the recall of a specific traumatic event. ^2^ Matches six items from the Mood and Anxiety Symptom Questionnaire (MASQ). ^3^ UK Biobank implementation*

**Table s5.** Overview of the UK Biobank fields queried in the current study.

|  | |
| --- | --- |
| **Category** | **UK Biobank Field ID** |
| **Sleep** | 1200 |
| **Neuroticism** | 1920, 1930, 1940, 1950, 1960, 1970, 1980, 1990, 2000, 2010, 2020, 2030 |
| **GAD-7** | 20506, 20509, 20520, 20515, 20516, 20505, 20512 |
| **Anxiety** | 20421, 20420, 20425, 20542, 20538, 20543, 20541, 20540, 20539, 20537, 20426, 20423, 20429, 20419, 20422, 20417, 20427 |
| **PCL-S** | 20497, 20498, 20495, 20496, 20494, 20508 |
| **PHQ-9** | 20514, 20510, 20517, 20519, 20511, 20507, 20508, 20518, 20513 |
| **Other** | 20428, 20549, 20550, 20418 |

| **Table s6.** R version overview. | | |
| --- | --- | --- |
| **Package** | **Loaded version** | **Date** |
| **bootnet** | 1.6.0 | 2024-02-21 |
| **corrplot** | 0.92 | 2021-11-18 |
| **dplyr** | 1.1.4 | 2023-11-17 |
| **forcats** | 1.0.0 | 2023-01-29 |
| **ggplot2** | 3.5.1 | 2024-04-23 |
| **ggridges** | 0.5.6 | 2024-01-23 |
| **kableExtra** | 1.4.0 | 2024-01-24 |
| **lavaan** | 0.6-18 | 2024-06-07 |
| **lubridate** | 1.9.3 | 2023-09-27 |
| **psych** | 2.4.3 | 2024-03-18 |
| **purrr** | 1.0.2 | 2023-08-10 |
| **readr** | 2.1.4 | 2023-02-10 |
| **rstatix** | 0.7.2 | 2023-02-01 |
| **stringr** | 1.5.1 | 2023-11-14 |
| **tableone** | 0.13.2 | 2022-04-15 |
| **tibble** | 3.2.1 | 2023-03-20 |
| **tidyr** | 1.3.1 | 2024-01-24 |
| **tidyverse** | 2.0.0 | 2023-02-22 |
| **qgraph** | 1.9.8 | 2023-11-03 |
| **semTools** | 0.5.7 | 2025-03-12 |
| **Stats** | 4.3.3 | 2024-03-01 |


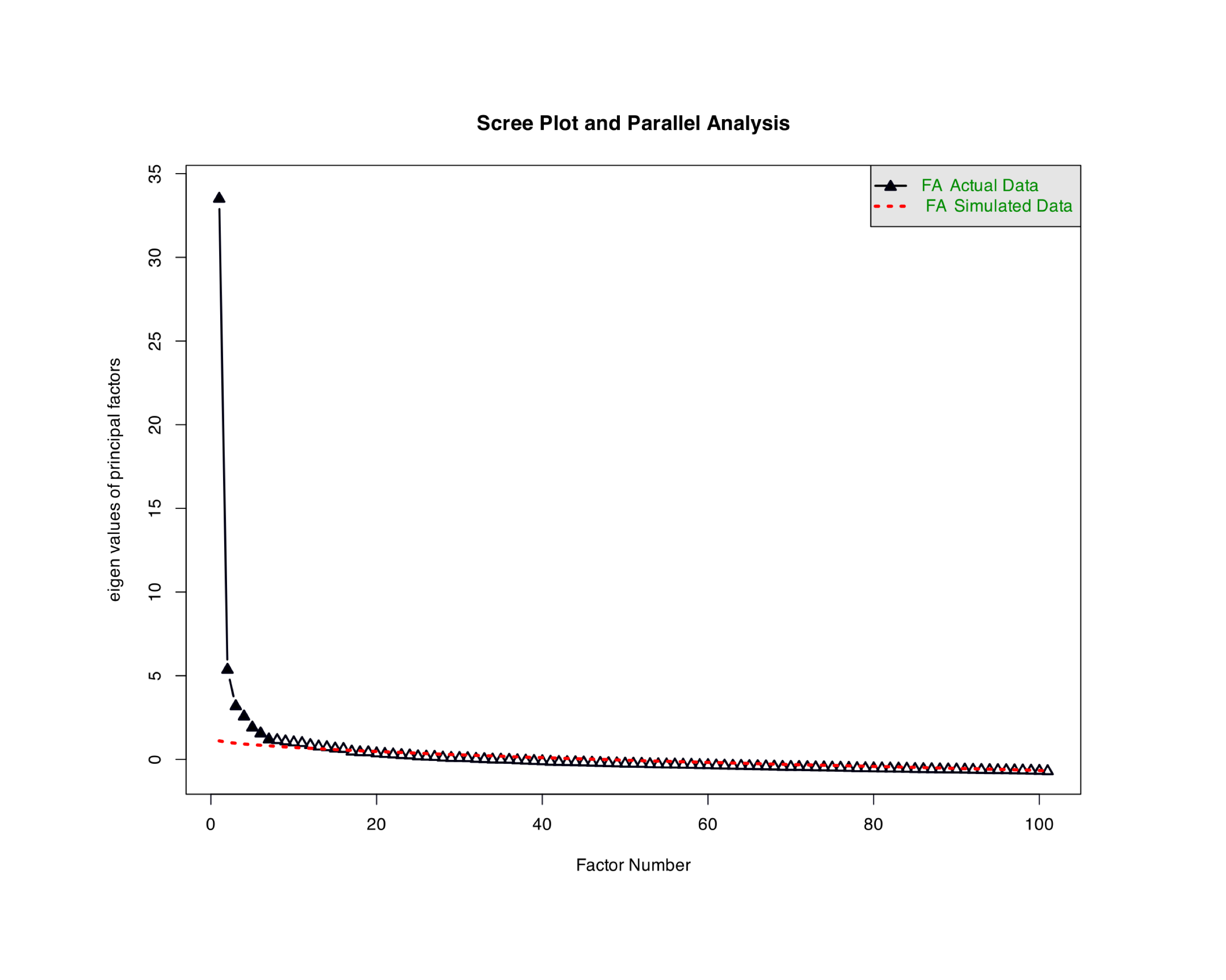


**Figure s1.** Scree plot of the exploratory factor analysis of the hyperarousal items.

*Closed triangles show the seven retained factors. Residual factors are shown as open dots to highlight how eigenvalues level off. Horizontal line shows an eigen value of one.*

**Table s7.** Factor loadings of the exploratory factor analysis.

|  | **Item** | **Anxious** | **Somatic** | **Sensitive** | **Sleep-**  **related** | **Irritable** | **Vigilant** | **Sudomotor** |
| --- | --- | --- | --- | --- | --- | --- | --- | --- |
| **STICSA** | I feel agonized over my problems. | **0.82** | 0.02 | -0.09 | 0.17 | -0.02 | -0.02 | -0.05 |
| **STICSA *** | I think that the worst will happen. | **0.81** | 0.02 | -0.02 | -0.07 | -0.14 | 0.08 | 0.03 |
| **PANAS *** | Afraid | **0.80** | 0.05 | 0.09 | 0.02 | -0.16 | 0.10 | 0.05 |
| **STICSA** | I picture some future misfortune. | **0.80** | 0.02 | -0.11 | -0.01 | 0.01 | 0.07 | -0.03 |
| **PANAS** | Scared | **0.79** | 0.05 | 0.09 | 0.06 | -0.17 | 0.08 | 0.04 |
| **STICSA *** | I feel like I’m missing out on things because I can’t  make up my mind soon enough. | **0.71** | -0.11 | -0.01 | -0.12 | 0.13 | 0.02 | 0.08 |
| **NQ** | Feelings of anxiety | **0.67** | 0.31 | 0.10 | 0.04 | -0.17 | 0.00 | -0.05 |
| **STICSA** | I can’t get some thought out of my mind. | **0.65** | -0.05 | -0.04 | **0.34** | 0.09 | -0.06 | -0.06 |
| **STICSA** | I worry that I cannot control my thoughts as well  as I would like to. | **0.64** | 0.05 | -0.08 | 0.30 | 0.01 | 0.01 | 0.00 |
| **N12 *** | Are you often troubled by feelings of guilt? | **0.63** | -0.16 | 0.09 | 0.06 | 0.13 | -0.10 | 0.02 |
| **PANAS** | Guilty | **0.62** | -0.15 | -0.04 | 0.14 | 0.12 | 0.01 | 0.08 |
| **PANAS** | Distressed | **0.58** | 0.12 | 0.01 | 0.12 | -0.03 | 0.10 | 0.00 |
| **HS** | When things go wrong, I tend to get depressed. | **0.58** | -0.12 | 0.19 | 0.01 | 0.18 | 0.13 | -0.17 |
| **HS** | I take a long time to make decisions. | **0.58** | -0.14 | 0.28 | -0.09 | -0.06 | 0.03 | 0.02 |
| **PANAS** | Ashamed | **0.57** | -0.06 | 0.01 | 0.07 | 0.07 | 0.04 | 0.15 |
| **STICSA** | I keep busy to avoid uncomfortable thoughts. | **0.56** | -0.04 | 0.01 | 0.21 | 0.01 | 0.11 | -0.01 |
| **N12** | Are you a worrier? | **0.56** | -0.15 | 0.24 | 0.07 | 0.22 | -0.09 | -0.07 |
| **N12** | Do you often feel lonely? | **0.54** | -0.06 | -0.10 | -0.05 | 0.20 | 0.13 | 0.04 |
| **PANAS** | Nervous | **0.52** | 0.17 | 0.09 | 0.11 | 0.00 | -0.07 | 0.16 |
| **N12** | Do you suffer from 'nerves'? | **0.52** | 0.22 | 0.26 | -0.21 | 0.09 | -0.02 | 0.03 |
| **STICSA** | I cannot concentrate without irrelevant thoughts  intruding | **0.51** | 0.02 | -0.04 | 0.14 | 0.17 | 0.08 | 0.07 |
| **N12** | Would you call yourself a nervous person? | **0.51** | 0.18 | **0.36** | -0.19 | 0.05 | -0.15 | 0.09 |
| **BAI** | Fear of dying | **0.50** | 0.22 | -0.11 | -0.14 | -0.03 | 0.09 | 0.06 |
| **HS** | I take things personally. | **0.47** | -0.04 | 0.27 | -0.07 | 0.28 | -0.07 | -0.01 |
| **HS** | I tend to anticipate problems. | **0.44** | -0.04 | **0.40** | 0.03 | 0.11 | -0.11 | -0.12 |
| **STICSA** | I have trouble remembering things. | **0.43** | 0.06 | -0.07 | 0.13 | 0.04 | 0.05 | 0.17 |
| **NQ** | Feeling tense | **0.43** | 0.28 | 0.12 | 0.19 | 0.03 | -0.10 | 0.00 |
| **STICSA** | I have butterflies in the stomach. | **0.42** | 0.32 | 0.00 | 0.09 | -0.02 | -0.02 | 0.00 |
| **HADS** | I feel restless as I have to be on the move | **0.35** | 0.01 | -0.04 | 0.27 | 0.11 | 0.05 | 0.00 |
| **PCL5** | Taking too many risks or doing things that  could cause you harm? | **0.34** | 0.09 | **-0.34** | 0.08 | 0.24 | 0.25 | -0.07 |
| **HS** | I am well-organized. | **-0.33** | -0.06 | 0.11 | -0.03 | -0.18 | -0.10 | 0.08 |
| **HS** | My mind is always going. | 0.31 | -0.04 | 0.31 | 0.28 | 0.01 | -0.05 | -0.05 |
| **N12** | Do you ever feel 'just miserable' for no reason? | 0.24 | 0.06 | 0.11 | 0.01 | 0.24 | 0.12 | -0.03 |
| **NQ *** | Tight feelings in the chest | 0.02 | **0.88** | 0.04 | -0.06 | -0.10 | 0.00 | -0.16 |
| **NQ *** | Palpitations | 0.00 | **0.84** | 0.13 | -0.01 | -0.05 | -0.20 | -0.03 |
| **NQ** | Chest pain | -0.02 | **0.80** | 0.07 | -0.04 | -0.03 | -0.12 | -0.20 |
| **PSAS** | Heart racing, pounding or beating irregularly | 0.02 | **0.77** | 0.01 | 0.03 | 0.00 | -0.15 | -0.02 |
| **STICSA** | My heart beats fast. | -0.01 | **0.73** | 0.07 | -0.10 | 0.07 | -0.12 | 0.12 |
| **PSAS** | Shortness of breath or labored breathing | 0.13 | **0.72** | -0.11 | 0.00 | 0.01 | 0.05 | -0.05 |
| **NQ *** | Faster/deeper breathing | 0.17 | **0.63** | 0.13 | -0.03 | 0.04 | -0.03 | -0.01 |
| **NQ *** | Tingling fingers | -0.18 | **0.63** | -0.05 | -0.01 | 0.04 | 0.11 | 0.11 |
| **NQ** | Unable to breathe deeply | -0.04 | **0.60** | 0.16 | 0.10 | 0.00 | 0.06 | -0.03 |
| **NQ** | Dizzy spells | 0.00 | **0.53** | 0.04 | -0.08 | 0.05 | 0.05 | 0.19 |
| **BAI** | Unsteady | 0.20 | **0.51** | -0.10 | -0.25 | -0.04 | 0.14 | 0.21 |
| **STICSA** | I feel dizzy. | 0.13 | **0.47** | -0.07 | -0.13 | 0.02 | 0.13 | 0.31 |
| **NQ** | Stiff fingers or arms | -0.30 | **0.45** | 0.05 | 0.14 | 0.05 | 0.20 | 0.09 |
| **STICSA *** | My arms and legs feel stiff. | 0.04 | **0.44** | -0.05 | 0.06 | 0.05 | 0.09 | 0.09 |
| **NQ** | Bloated feeling in the stomach | -0.19 | **0.44** | 0.15 | 0.04 | 0.16 | 0.01 | 0.18 |
| **PSAS** | Have stomach upset (knot or nervous feeling  in stomach, heartburn, nausea, gas etc.) | -0.01 | **0.41** | -0.04 | 0.19 | 0.17 | -0.07 | 0.09 |
| **NQ** | Blurred vision | -0.03 | **0.41** | -0.01 | 0.06 | 0.11 | 0.11 | 0.22 |
| **BAI** | Feeling of choking | **0.34** | **0.41** | -0.19 | 0.12 | -0.19 | 0.06 | 0.01 |
| **NQ** | Cold hands or feet | -0.14 | **0.40** | 0.21 | 0.09 | 0.01 | 0.07 | -0.18 |
| **PSAS** | A jittery, nervous feeling in your body | **0.39** | **0.39** | -0.04 | 0.32 | -0.07 | -0.07 | -0.02 |
| **NQ** | Tight feelings around the mouth | -0.01 | **0.35** | 0.01 | 0.17 | -0.05 | 0.19 | 0.13 |
| **PSAS** | Dry feeling in mouth or throat | -0.02 | 0.30 | -0.05 | 0.15 | 0.00 | 0.01 | 0.28 |
| **HSPS** | I feel uncomfortable by loud noises | -0.27 | 0.00 | **0.65** | 0.06 | 0.05 | **0.41** | 0.02 |
| **HSPS *** | I tend to be more sensitive to pain | -0.02 | 0.21 | **0.65** | -0.12 | -0.08 | -0.01 | 0.02 |
| **APS *** | I tend to remain excited or moved for a long period  of time after seeing a good movie. | 0.08 | 0.05 | **0.64** | -0.10 | 0.02 | 0.00 | 0.03 |
| **HSPS** | Being very hungry creates a strong reaction in me,  disrupting my concentration or mood | -0.24 | -0.02 | **0.63** | -0.06 | 0.16 | -0.07 | 0.21 |
| **HSPS** | I am bothered by intense stimuli, like loud noises  or chaotic scenes | -0.23 | -0.06 | **0.62** | 0.06 | 0.07 | **0.48** | 0.01 |
| **HSPS *** | I startle easily | 0.15 | 0.03 | **0.55** | -0.05 | -0.03 | 0.19 | 0.07 |
| **HSPS** | I get annoyed when people try to get me to do  too many things at once | 0.03 | -0.18 | **0.54** | -0.09 | 0.16 | 0.27 | 0.04 |
| **HSPS** | I am deeply moved by the arts or music | -0.07 | 0.10 | **0.53** | 0.02 | 0.01 | 0.10 | -0.16 |
| **APS** | I get excited easily. | 0.02 | 0.00 | **0.47** | -0.04 | 0.28 | -0.11 | 0.12 |
| **HSPS** | I am conscientious | 0.20 | -0.06 | **0.47** | -0.08 | -0.10 | 0.22 | -0.04 |
| **HSPS *** | As a child, my parents or teachers seem to see  me as sensitive or shy | 0.09 | 0.06 | **0.44** | 0.03 | -0.11 | 0.08 | -0.04 |
| **FIRST** | After watching a frightening movie or TV show | -0.06 | 0.06 | **0.43** | 0.12 | -0.06 | -0.03 | 0.05 |
| **HS** | I am a cautious person. | 0.31 | -0.10 | **0.42** | -0.05 | -0.21 | 0.08 | 0.14 |
| **N12** | Do you worry too long after an embarrassing  experience? | 0.27 | 0.02 | **0.42** | 0.05 | 0.21 | -0.11 | -0.02 |
| **HSPS** | I am aware of subtleties in my environment | 0.00 | -0.07 | **0.39** | 0.26 | -0.05 | 0.27 | 0.01 |
| **HS** | I get tearful easily. | 0.31 | 0.06 | **0.39** | -0.10 | 0.02 | -0.08 | -0.10 |
| **HSPS** | I try hard to avoid making mistakes or  forgetting things | **0.35** | -0.09 | **0.38** | 0.02 | 0.01 | 0.23 | -0.08 |
| **FIRST** | Before having to speak in public | 0.17 | -0.05 | 0.32 | 0.18 | -0.05 | -0.08 | 0.12 |
| **PSAS** | Can’t shut off your thoughts | 0.17 | -0.02 | -0.07 | **0.84** | 0.11 | -0.16 | 0.01 |
| **PSAS *** | Review or ponder events of the day | 0.15 | -0.05 | 0.00 | **0.75** | 0.15 | -0.18 | -0.10 |
| **PSAS *** | Being mentally alert, active | 0.00 | 0.01 | 0.02 | **0.74** | 0.05 | -0.03 | 0.02 |
| **PSAS** | Thoughts keep running through your head | 0.24 | -0.02 | -0.04 | **0.72** | 0.10 | -0.22 | 0.03 |
| **PCL5 *** | Trouble falling or staying asleep? | 0.17 | 0.12 | -0.18 | **0.66** | -0.10 | 0.01 | -0.01 |
| **HS *** | I have trouble falling asleep. | -0.02 | 0.10 | -0.05 | **0.64** | -0.11 | 0.01 | -0.03 |
| **PSAS** | Being distracted by sounds, noise in the environment  (e.g. ticking of clock, house noises, traffic) | -0.20 | 0.05 | 0.07 | **0.54** | 0.18 | 0.09 | 0.00 |
| **FIRST** | After an argument | 0.15 | -0.13 | 0.32 | **0.50** | -0.05 | -0.05 | 0.11 |
| **FIRST** | After having a bad day at work | 0.18 | -0.15 | 0.26 | **0.45** | 0.00 | -0.10 | 0.16 |
| **N12 *** | Are you an irritable person? | 0.06 | 0.02 | 0.06 | -0.15 | **0.83** | -0.09 | 0.01 |
| **DASS21 *** | I felt that I was rather touchy | 0.15 | 0.02 | 0.02 | 0.02 | **0.65** | 0.09 | 0.00 |
| **PANAS *** | Irritable | -0.04 | 0.05 | -0.04 | 0.17 | **0.63** | 0.09 | 0.06 |
| **PANAS *** | Hostile | 0.13 | -0.09 | -0.05 | 0.13 | **0.61** | 0.15 | -0.04 |
| **N12** | Do you often feel 'fed-up'? | 0.16 | 0.02 | -0.10 | 0.14 | **0.59** | -0.02 | -0.03 |
| **DASS21** | I was intolerant of anything that kept me from  getting on with what I was doing | 0.17 | -0.01 | 0.10 | 0.01 | **0.51** | 0.14 | -0.01 |
| **N12** | Are your feelings easily hurt? | 0.30 | 0.07 | 0.14 | 0.02 | **0.47** | -0.07 | -0.16 |
| **APS** | I am a calm person. | 0.16 | 0.20 | -0.07 | 0.02 | 0.32 | 0.05 | -0.07 |
| **BHS *** | I notice that when I am in public or new places, I need to scan the crowd or surroundings. | 0.19 | 0.01 | 0.12 | -0.15 | 0.11 | **0.74** | -0.17 |
| **BHS *** | When I am in public, I feel overwhelmed because  I cannot keep track of everything going on around me. | 0.25 | 0.01 | 0.14 | -0.21 | 0.12 | **0.70** | -0.05 |
| **BHS** | I feel that if I don’t stay alert and watchful,  something bad will happen. | **0.52** | -0.04 | 0.12 | -0.08 | -0.09 | **0.56** | -0.10 |
| **BHS** | When I am outside, I think ahead about what  I would do (or where I would go) if someone  would try to surprise or harm me. | 0.32 | 0.11 | 0.05 | -0.25 | 0.16 | **0.36** | -0.03 |
| **HSPS** | When people are uncomfortable in a physical environment  I tend to know what needs to be done to make it more comfortable (like changing the lighting or the seating) | 0.00 | 0.03 | 0.21 | 0.15 | -0.16 | 0.25 | -0.01 |
| **HS** | I am slow to awaken mornings. | 0.02 | 0.11 | 0.10 | 0.06 | -0.01 | 0.23 | -0.04 |
| **BAI *** | Feeling hot | 0.00 | 0.06 | 0.14 | 0.06 | -0.07 | -0.13 | **0.69** |
| **BAI *** | Hot/cold sweats | 0.09 | 0.20 | 0.10 | 0.01 | -0.12 | -0.15 | **0.66** |
| **PSAS *** | Perspiration in palms of your hands or other parts of your body | -0.03 | 0.19 | 0.00 | 0.04 | 0.08 | -0.08 | **0.63** |
| **STICSA *** | My face feels hot. | 0.15 | 0.22 | 0.01 | -0.06 | 0.05 | -0.02 | **0.53** |
| **STICSA** | My palms feel clammy. | **0.44** | 0.22 | -0.10 | -0.21 | -0.04 | 0.00 | **0.46** |

*Factor loadings of the exploratory factor analysis after reducing the most collinear items.* *Factor loadings >= |0.32| are in bold. * selected items to represent the dimensions of hyperarousal. STICSA, State-Trait Inventory for Cognitive and Somatic Anxiety; PANAS, Positive and Negative Affect Schedule; NQ, Nijmegen Hyperventilation Questionnaire; N12, Eysenck Neuroticism 12; HS, Hyperarousal scale; BHS, Brief Hypervigilance Scale; BAI, Beck Anxiety Inventory; PSAS, Pre-sleep arousal scale; HSPS, High Sensitive Person Scale; HADS, Hospital Anxiety and Depression Scale; PCL5, PTSD checklist for dsm-5; FIRST, Ford Insomnia Response to Stress Test; DASS21, Depression, Anxiety and Stress Scale; APS, Arousal predisposition scale.*


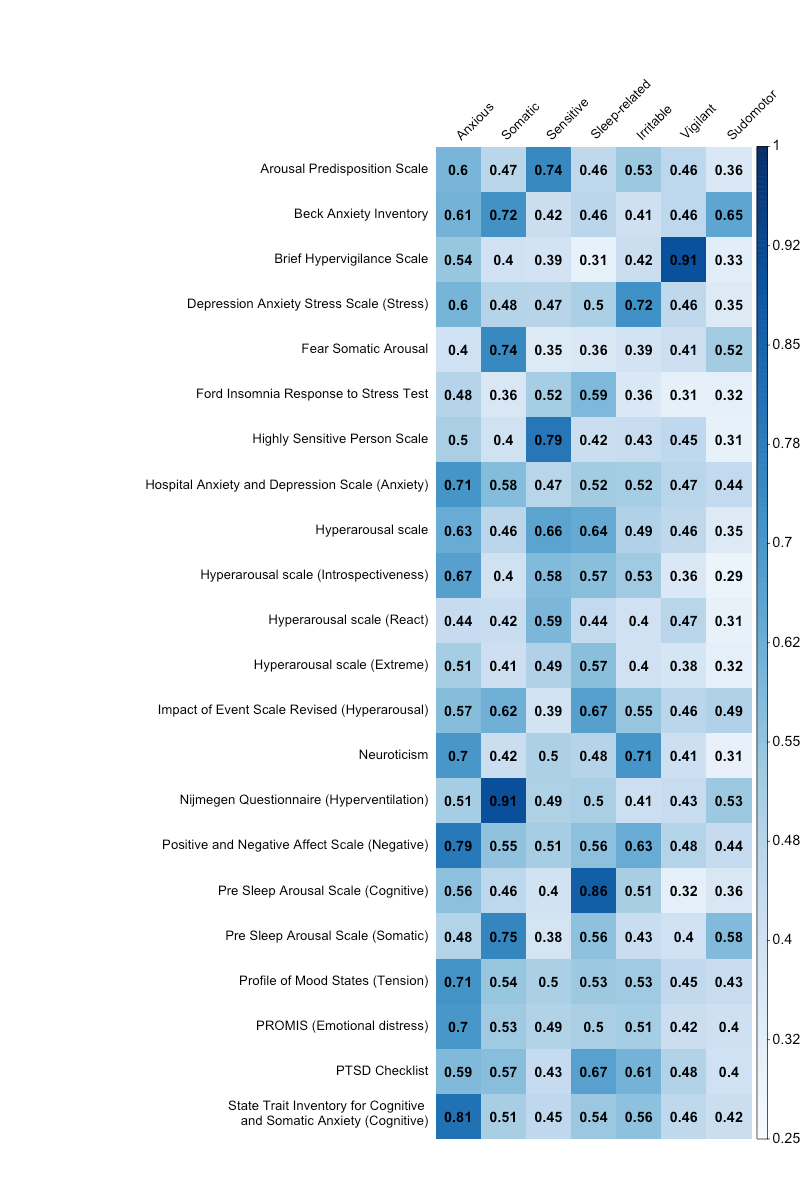
**Figure s2.** Correlation matrix of the hyperarousal factor scores and the original hyperarousal-related scales. *Subscales are indicated with brackets. Note: color scale range was adjusted to increase visual discrimination of correlation values.*


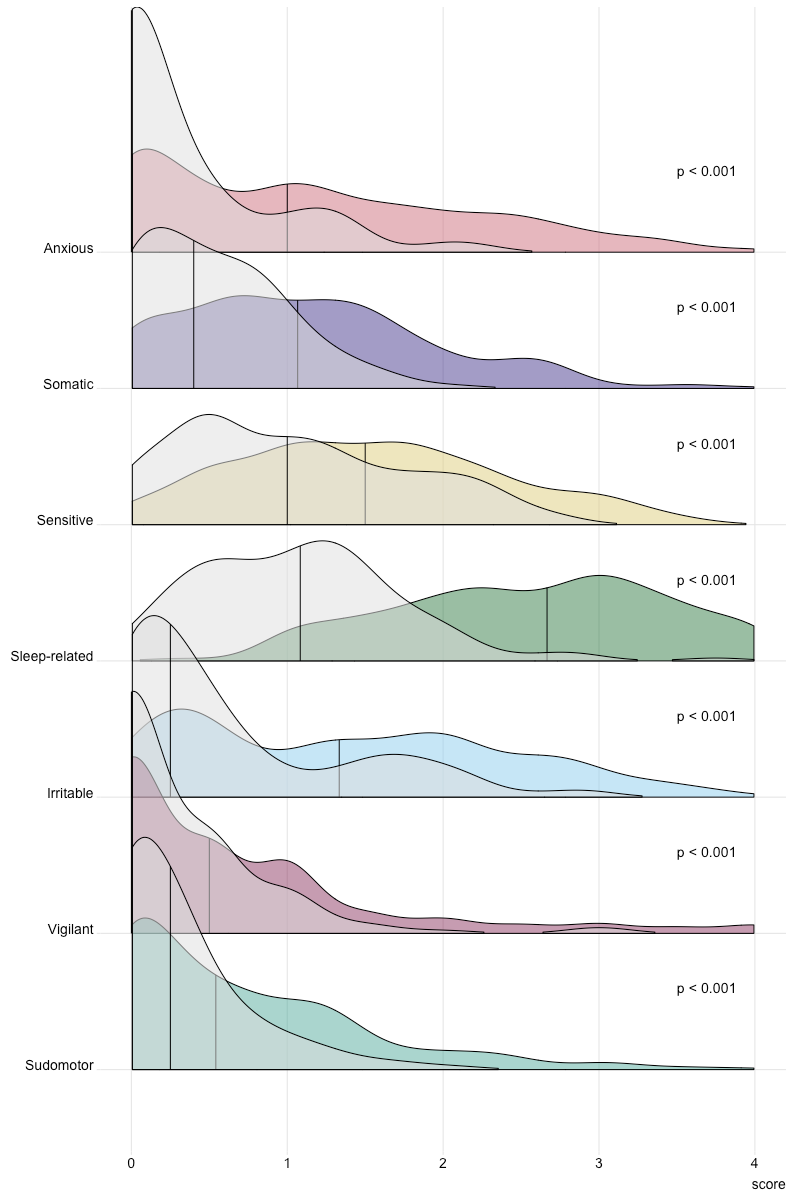


**Figure s3**. Distribution of the symptom severity scores in the seven dimensions of hyperarousal assessed with the THDQ in 467 participants.

*Grey distributions show the hyperarousal dimension scores of people unlikely to have any mental disorder diagnosis (n = 135) according to scores below the cut-off on the ISI, ASRS and all subscales of the RMT20. The colored distributions represent people who do cross at least one cut-off for a probable mental disorder diagnosis (n = 332). Vertical solid line indicates the median score within each group. Note that the median for Anxious and Vigilant hyperarousal in people unlikely to have any mental disorder diagnosis is zero. Groups were compared using Wilcoxon tests and p-values are shown. RMT20, Rapid Measurement Toolkit-20; ISI, Insomnia Severity Index; ASRS, Adult ADHD Self Report Scale.*

**Table s8.** Contribution of each hyperarousal dimension to the severity of symptoms characterizing each disorder.

|  |  | **ID** | **MDD** | **GAD** | **SAD** | **PD** | **PTSD** | **ADHD** |
| --- | --- | --- | --- | --- | --- | --- | --- | --- |
| Anxious | β (SE) | 0.04 (0.04) | 0.43 (0.04) | **0.51 (0.04)** | 0.32 (0.05) | 0.37 (0.05) | 0.31 (0.05) | 0.25 (0.05) |
|  | CI | -0.05 - 0.12 | 0.34 - 0.51 | **0.44 - 0.59** | 0.23 - 0.41 | 0.27 - 0.46 | 0.22 - 0.41 | 0.15 - 0.36 |
| Somatic | β (SE) | 0.08 (0.04) | 0.10 (0.04) | 0.12 (0.04) | 0.09 (0.04) | **0.22 (0.05)** | 0.10 (0.05) | 0.07 (0.05) |
|  | CI | 0.00 - 0.16 | 0.02 - 0.18 | 0.05 - 0.20 | 0.01 - 0.18 | **0.13 - 0.32** | 0.01 - 0.20 | -0.03 - 0.17 |
| Sensitive | β (SE) | -0.03 (0.04) | -0.10 (0.04) | 0.04 (0.04) | **0.18 (0.04)** | 0.00 (0.04) | 0.00 (0.05) | 0.01 (0.05) |
|  | CI | -0.11 - 0.04 | -0.18 - -0.02 | -0.03 - 0.10 | **0.10 - 0.26** | -0.09 - 0.09 | -0.09 - 0.09 | -0.09 - 0.10 |
| Sleep-related | β (SE) | **0.70 (0.04)** | 0.20 (0.04) | 0.23 (0.03) | 0.11 (0.04) | 0.07 (0.04) | 0.06 (0.04) | 0.14 (0.05) |
|  | CI | **0.62 - 0.77** | 0.12 - 0.28 | 0.16 - 0.30 | 0.03 - 0.19 | -0.02 - 0.15 | -0.02 - 0.15 | 0.04 - 0.23 |
| Irritable | β (SE) | 0.09 (0.04) | **0.19 (0.04)** | 0.10 (0.03) | 0.10 (0.04) | -0.07 (0.04) | 0.05 (0.04) | 0.14 (0.05) |
|  | CI | 0.01 - 0.16 | **0.11 - 0.27** | 0.03 - 0.17 | 0.02 - 0.18 | -0.16 - 0.01 | -0.04 - 0.13 | 0.04 - 0.23 |
| Vigilant | β (SE) | -0.03 (0.04) | 0.07 (0.04) | 0.01 (0.03) | 0.20 (0.04) | 0.19 (0.04) | **0.25 (0.04)** | 0.08 (0.05) |
|  | CI | -0.10 - 0.04 | 0.00 - 0.15 | -0.05 - 0.08 | 0.12 - 0.27 | 0.10 - 0.27 | **0.16 - 0.33** | -0.01 - 0.17 |
| Sudomotor | β (SE) | 0.04 (0.04) | 0.05 (0.04) | 0.02 (0.03) | 0.06 (0.04) | 0.06 (0.04) | **0.12 (0.04)** | 0.06 (0.05) |
|  | CI | -0.04 - 0.11 | -0.03 - 0.13 | -0.05 - 0.08 | -0.02 - 0.14 | -0.03 - 0.14 | **0.04 - 0.21** | -0.03 - 0.16 |

*Standardized beta-coefficients, standard error and 95% confidence interval from the multiple regression analyses evaluating the contribution of each hyperarousal dimension to the severity of symptoms characterizing each disorder. Significant coefficients are underlined. Note that different hyperarousal dimensions show the largest effect size (in bold) in association with different severities of symptoms characterizing each disorder (left-right): Anxious with GAD, Somatic with PD, Sensitive with SAD, Sleep with ID, Irritable with MDD, Vigilant and Sudomotor with PTSD.*

**Table s9.** Complementary regression models for each disorder and dimension independently.

|  |  | **ID** | **MDD** | **GAD** | **SAD** | **PD** | **PTSD** | **ADHD** |
| --- | --- | --- | --- | --- | --- | --- | --- | --- |
| Anxious | β (SE) | 0.36 (0.05) | 0.63 (0.04) | **0.72 (0.03)** | 0.61 (0.04) | 0.55 (0.04) | 0.54 (0.04) | 0.45 (0.04) |
|  | CI | 0.27 - 0.45 | 0.56 - 0.71 | **0.66 - 0.79** | 0.53 - 0.68 | 0.47 - 0.63 | 0.47 - 0.62 | 0.37 - 0.54 |
| Somatic | β (SE) | 0.42 (0.04) | 0.43 (0.04) | **0.48 (0.04)** | 0.44 (0.04) | 0.47 (0.04) | 0.42 (0.04) | 0.37 (0.04) |
|  | CI | 0.33 - 0.51 | 0.34 - 0.51 | **0.40 - 0.56** | 0.36 - 0.53 | 0.38 - 0.55 | 0.33 - 0.50 | 0.28 - 0.46 |
| Sensitive | β (SE) | 0.25 (0.05) | 0.31 (0.05) | 0.44 (0.04) | **0.50 (0.04)** | 0.34 (0.05) | 0.34 (0.05) | 0.31 (0.05) |
|  | CI | 0.16 - 0.35 | 0.22 - 0.40 | 0.36 - 0.53 | **0.42 - 0.59** | 0.25 - 0.43 | 0.25 - 0.43 | 0.22 - 0.40 |
| Sleep-related | β (SE) | **0.76 (0.03)** | 0.47 (0.04) | 0.54 (0.04) | 0.41 (0.04) | 0.35 (0.04) | 0.36 (0.04) | 0.35 (0.04) |
|  | CI | **0.70 - 0.82** | 0.39 - 0.55 | 0.46 - 0.61 | 0.33 - 0.50 | 0.26 - 0.43 | 0.28 - 0.45 | 0.27 - 0.44 |
| Irritable | β (SE) | 0.37 (0.05) | **0.50 (0.04)** | 0.48 (0.04) | 0.45 (0.04) | 0.27 (0.05) | 0.36 (0.04) | 0.37 (0.04) |
|  | CI | 0.28 - 0.46 | **0.42 - 0.58** | 0.40 - 0.56 | 0.37 - 0.54 | 0.18 - 0.36 | 0.27 - 0.44 | 0.29 - 0.46 |
| Vigilant | β (SE) | 0.21 (0.05) | 0.36 (0.04) | 0.35 (0.04) | **0.48 (0.04)** | 0.42 (0.04) | 0.47 (0.04) | 0.30 (0.05) |
|  | CI | 0.12 - 0.30 | 0.27 - 0.45 | 0.27 - 0.44 | **0.40 - 0.56** | 0.34 - 0.51 | 0.39 - 0.55 | 0.22 - 0.39 |
| Sudomotor | β (SE) | 0.32 (0.05) | 0.33 (0.04) | 0.34 (0.04) | 0.36 (0.04) | 0.33 (0.04) | **0.37 (0.04)** | 0.27 (0.05) |
|  | CI | 0.23 - 0.41 | 0.25 - 0.42 | 0.26 - 0.43 | 0.27 - 0.44 | 0.25 - 0.42 | **0.29 - 0.46** | 0.18 - 0.36 |

*Standardized beta-coefficients, standard error and 95% confidence interval from evaluating the contribution of each individual hyperarousal dimension to the severity of symptoms characterizing each disorder using separate regression analyses for each dimension and disorder with age and sex as covariates. Largest effect sizes for each dimension (left-right) are in bold. All coefficients were significant.*

**Table s10.** Connection strength of the significant

| **Node 1** | **Node 2** | **Connection**  **strength** | **p-value** |
| --- | --- | --- | --- |
| ID | Sleep-related | 0.585 | 0.001 |
| GAD | Anxious | 0.315 | 0.001 |
| MDD | GAD | 0.303 | 0.001 |
| Somatic | Sudomotor | 0.283 | 0.001 |
| PTSD | Vigilant | 0.190 | 0.002 |
| PD | PTSD | 0.181 | 0.002 |
| MDD | Irritable | 0.173 | 0.001 |
| PD | Somatic | 0.171 | 0.003 |
| SAD | Vigilant | 0.163 | 0.002 |
| SAD | PD | 0.161 | 0.002 |
| Anxious | Sensitive | 0.150 | 0.004 |
| SAD | Sensitive | 0.150 | 0.001 |
| GAD | SAD | 0.134 | 0.005 |
| GAD | Sleep-related | 0.133 | 0.002 |
| MDD | PTSD | 0.132 | 0.015 |
| ADHD | Anxious | 0.125 | 0.011 |
| ADHD | Irritable | 0.124 | 0.008 |
| MDD | Anxious | 0.121 | 0.008 |
| PD | Anxious | 0.113 | 0.029 |
| GAD | PD | 0.111 | 0.006 |
| Somatic | Sensitive | 0.108 | 0.013 |
| MDD | SAD | 0.099 | 0.019 |
| ID | GAD | 0.070 | 0.050 |

*P-values were approximated using 2000 bootstraps of connection strengths.*


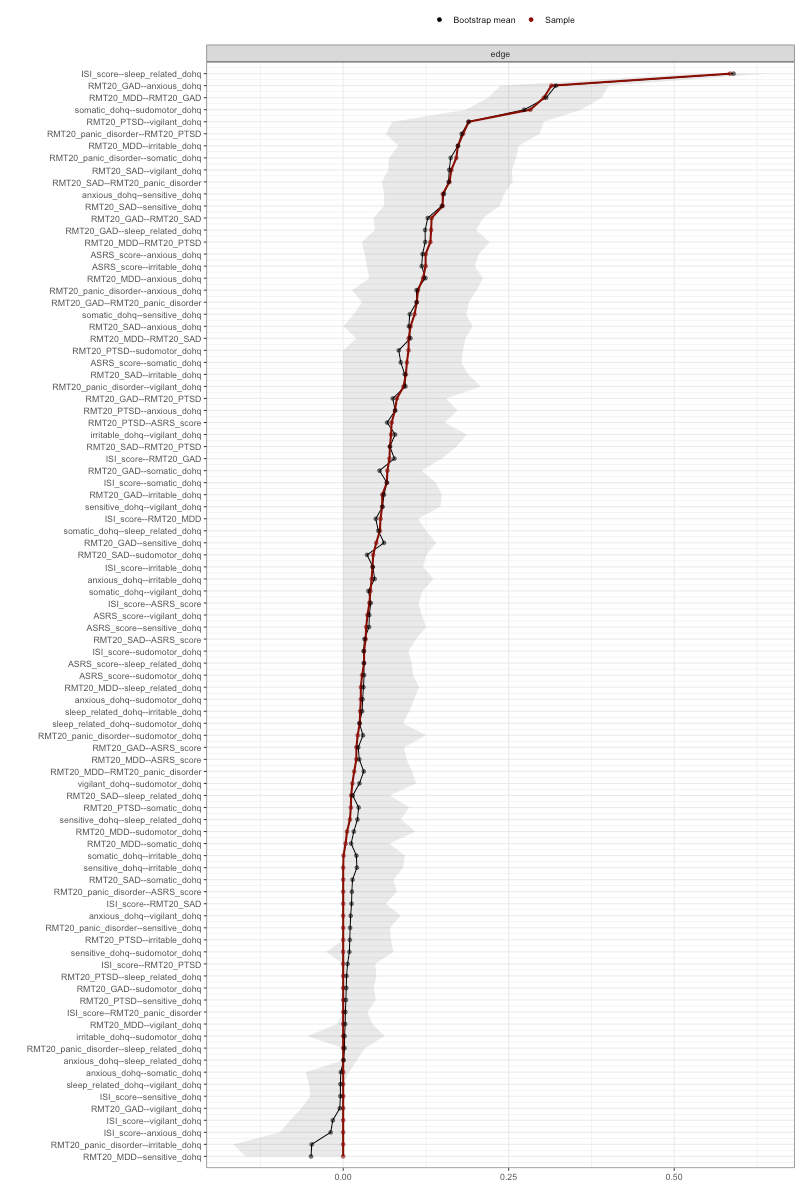


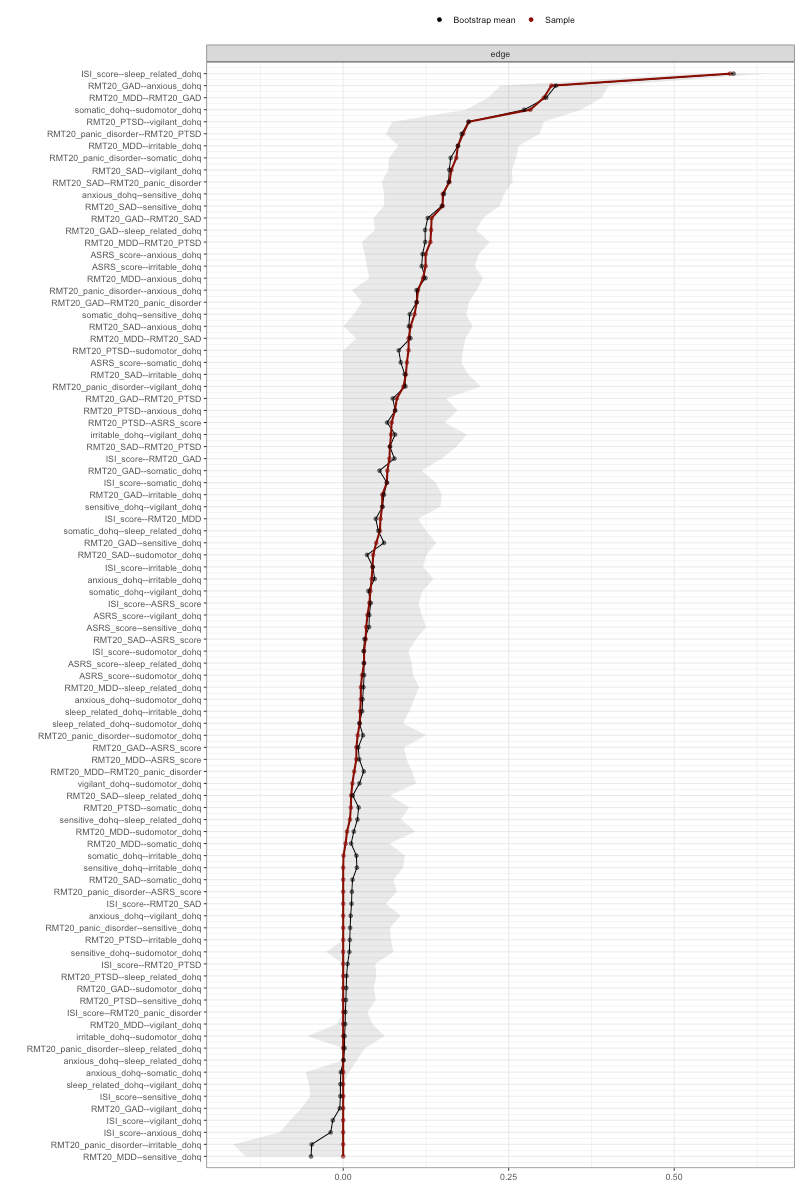


**Figure s4a**. Edge weight stability (figure continues on the next page)

*Non-parametric bootstrap was used to create 1000 samples to estimate the stability of the edge weights. Bootstrap mean and sample weight of each edge are shown with the 95% confidence interval in grey shade. The twenty-two edges of the sparse network (figure 2) are stable indicated by the confidence intervals without zero.*


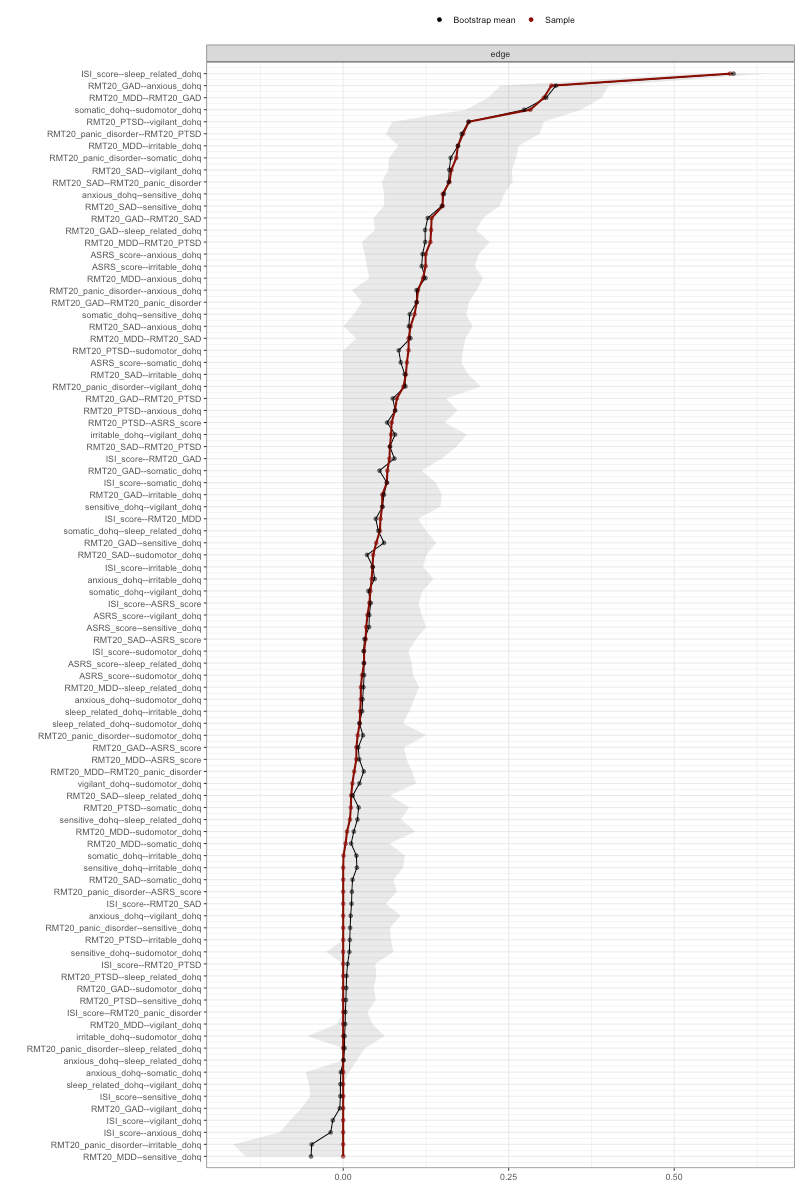

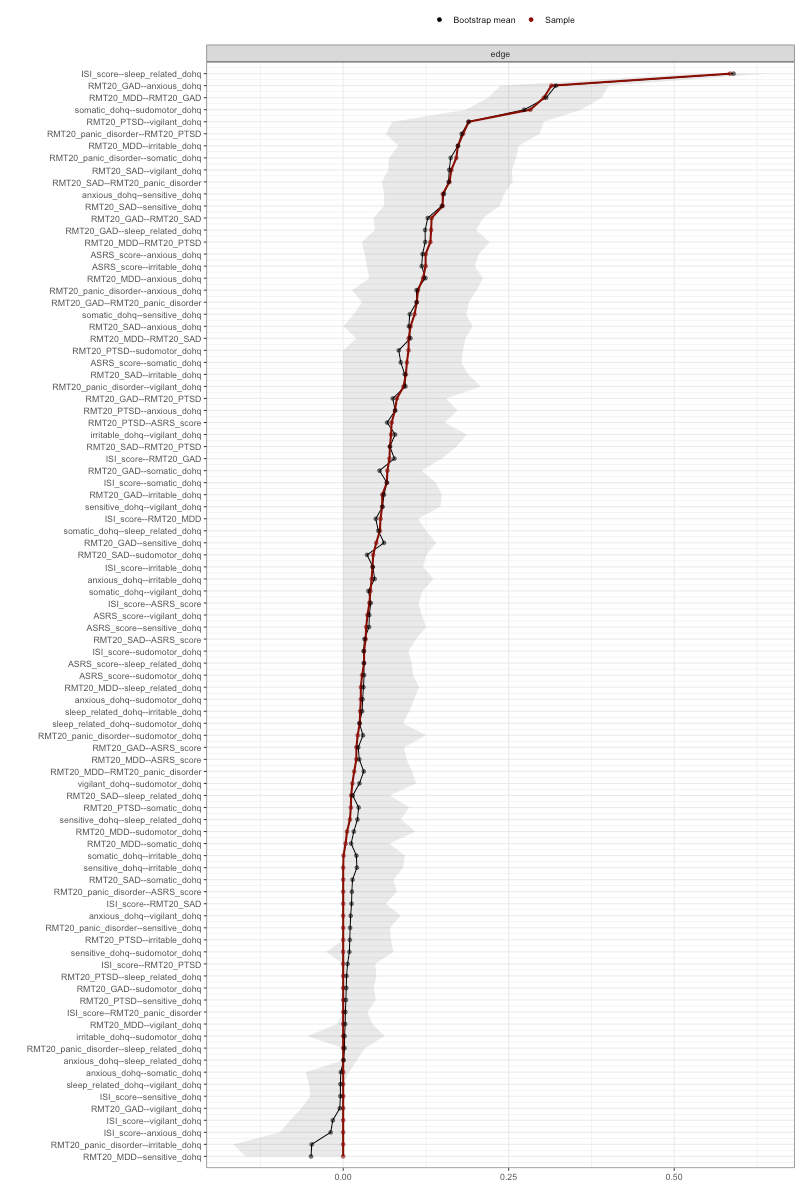


**Figure s4b**. Edge weight stability

*Non-parametric bootstrap was used to create 1000 samples to estimate the stability of the edge weights. Bootstrap mean and sample weight of each edge are shown with the 95% confidence interval in grey shade. The twenty-two edges of the sparse network (figure 2) are stable indicated by the confidence intervals without zero.*

**Table s11.** Demographics of the second sample.

|  | **All** | **Subset** |
| --- | --- | --- |
| **n** | 592 | 315 |
| **Age ^1^** | 61.0 (12.7) | 60.8 (12.8) |
| **Female ^2^** | 386 (65.2) | 238 (75.6) |

*Subset participated in sample 1 and 2. Demographic data were unavailable for n=72. ^1^ mean (SD), ^2^ n (%)*

**Table s12.** Sensitivity analysis of multiple regression analyses.

|  |  | **ID** | **MDD** | **GAD** | **SAD** | **PD** | **PTSD** | **ADHD** |
| --- | --- | --- | --- | --- | --- | --- | --- | --- |
| Anxious | β (SE) | 0.02 (0.04) | 0.42 (0.05) | **0.50 (0.04)** | 0.32 (0.05) | 0.38 (0.05) | 0.30 (0.05) | 0.26 (0.05) |
|  | CI | -0.06 - 0.11 | 0.33 - 0.51 | **0.42 - 0.58** | 0.23 - 0.41 | 0.28 - 0.47 | 0.20 - 0.40 | 0.15 - 0.37 |
| Somatic | β (SE) | 0.08 (0.04) | 0.10 (0.04) | 0.13 (0.04) | 0.09 (0.04) | **0.23 (0.05)** | 0.11 (0.05) | 0.07 (0.05) |
|  | CI | 0.00 - 0.16 | 0.01 - 0.18 | 0.05 - 0.20 | 0.00 - 0.18 | **0.14 - 0.32** | 0.02 - 0.20 | -0.04 - 0.17 |
| Sensitive | β (SE) | -0.03 (0.04) | -0.11 (0.04) | 0.03 (0.04) | **0.18 (0.04)** | 0.01 (0.04) | 0.00 (0.05) | -0.01 (0.05) |
|  | CI | -0.11 - 0.05 | -0.19 - -0.02 | -0.04 - 0.10 | **0.09 - 0.26** | -0.08 - 0.10 | -0.09 - 0.09 | -0.10 - 0.09 |
| Sleep-related | β (SE) | **0.70 (0.04)** | 0.19 (0.04) | 0.23 (0.03) | 0.10 (0.04) | 0.07 (0.04) | 0.05 (0.04) | 0.14 (0.05) |
|  | CI | **0.62 - 0.77** | 0.11 - 0.27 | 0.16 - 0.30 | 0.02 - 0.19 | -0.02 - 0.15 | -0.03 - 0.14 | 0.04 - 0.24 |
| Irritable | β (SE) | 0.08 (0.04) | **0.20 (0.04)** | 0.10 (0.03) | 0.10 (0.04) | -0.09 (0.04) | 0.05 (0.04) | 0.14 (0.05) |
|  | CI | 0.00 - 0.15 | **0.12 - 0.28** | 0.03 - 0.17 | 0.02 - 0.18 | -0.17 - 0.00 | -0.03 - 0.14 | 0.04 - 0.23 |
| Vigilant | β (SE) | -0.02 (0.04) | 0.07 (0.04) | 0.02 (0.03) | 0.19 (0.04) | 0.18 (0.04) | **0.24 (0.04)** | 0.09 (0.05) |
|  | CI | -0.09 - 0.05 | 0.00 - 0.15 | -0.05 - 0.08 | 0.11 - 0.27 | 0.09 - 0.26 | **0.16 - 0.33** | 0.00 - 0.19 |
| Sudomotor | β (SE) | 0.04 (0.04) | 0.04 (0.04) | 0.01 (0.04) | 0.05 (0.04) | 0.06 (0.04) | **0.11 (0.04)** | 0.06 (0.05) |
|  | CI | -0.04 - 0.12 | -0.04 - 0.12 | -0.06 - 0.08 | -0.03 - 0.13 | -0.03 - 0.15 | **0.02 - 0.20** | -0.03 - 0.16 |

*Standardized beta-coefficients, standard error and 95% confidence interval from evaluating the contribution of each individual hyperarousal dimension to the severity of symptoms characterizing each disorder using multiple regression analyses for each disorder with age, sex, years of education and income as covariate. Largest effect sizes for each dimension (left-right) are in bold. Significant coefficients are underlined.*

**Table s13** Demographics of UK Biobank.

|  | **UK Biobank** |
| --- | --- |
| n | 501,938 |
| Age at recruitment | 56.5 (8.09) |
| Female | 273,037 (54.4 %) |
| Age completed full education | 16.7 (2.33) |
| College or university degree | 229,569 (46.0 %) |

*Source: UK Biobank showcase visited 19-12-2025 (https://biobank.ctsu.ox.ac.uk/crystal/index.cgi)*

**Table s14.** Hyperarousal factor loadings of UK Biobank items.

| **UKB Field ID** | **Anxious** | **Somatic** | **Sensitive** | **Sleep-related** | **Irritable** | **Vigilant** | **Sudomotor** |
| --- | --- | --- | --- | --- | --- | --- | --- |
| **2030** | **0.87** | -0.30 | 0.24 | 0.03 | 0.10 | -0.21 | 0.00 |
| **20507** | **0.87** | -0.09 | -0.13 | -0.03 | 0.24 | -0.04 | -0.08 |
| **20512** | **0.81** | 0.10 | -0.13 | 0.06 | -0.17 | 0.16 | 0.01 |
| **20520** | **0.69** | 0.16 | -0.06 | 0.22 | 0.10 | -0.10 | -0.04 |
| **20509** | **0.68** | 0.07 | -0.06 | 0.28 | 0.12 | -0.03 | -0.12 |
| **2020** | **0.63** | 0.07 | -0.21 | -0.09 | 0.25 | 0.07 | -0.02 |
| **20510** | **0.58** | 0.15 | -0.24 | 0.13 | 0.17 | 0.07 | -0.06 |
| **1980** | **0.56** | -0.19 | 0.32 | 0.09 | 0.21 | -0.03 | -0.14 |
| **2010** | **0.48** | 0.25 | 0.16 | -0.14 | 0.11 | 0.10 | -0.03 |
| **20519** | 0.07 | **0.46** | -0.11 | 0.28 | 0.11 | 0.04 | -0.08 |
| **28696** | -0.10 | **0.45** | -0.04 | 0.19 | 0.13 | 0.09 | -0.08 |
| **2000** | 0.27 | -0.11 | **0.52** | 0.14 | 0.16 | -0.07 | -0.03 |
| **1200** | 0.00 | -0.11 | -0.21 | **1.04** | -0.07 | -0.04 | 0.05 |
| **28687** | 0.04 | -0.12 | -0.24 | **0.98** | -0.07 | 0.10 | 0.02 |
| **20517** | 0.09 | 0.03 | -0.19 | **0.87** | 0.02 | -0.08 | -0.04 |
| **28699** | 0.01 | 0.25 | -0.01 | **0.47** | 0.01 | 0.08 | 0.04 |
| **1940** | 0.15 | 0.01 | -0.08 | -0.30 | **1.11** | -0.23 | 0.03 |
| **20505** | -0.15 | 0.04 | -0.10 | 0.05 | **0.96** | 0.05 | 0.06 |
| **1960** | 0.26 | 0.01 | -0.21 | 0.09 | **0.64** | -0.02 | 0.00 |
| **1920** | 0.27 | 0.01 | 0.09 | -0.04 | **0.63** | -0.13 | -0.04 |
| **1950** | 0.31 | 0.05 | 0.09 | 0.03 | **0.55** | -0.11 | -0.15 |
| **28690** | -0.27 | -0.14 | 0.19 | -0.07 | 0.09 | -0.17 | **0.99** |

*Loadings with an absolute value of 0.32 or higher are in bold for clarity.*

**Transdiagnostic hyperarousal dimensions questionnaire (NL)**

Hieronder ziet u een aantal woorden en zinnen. Mensen verschillen in de mate waarin zo’n woord of zin beschrijft hoe zij zichzelf over het algemeen ervaren. Wilt u bij elk van de onderstaande woorden of zinnen aangeven in welke mate deze op uzelf van toepassing is?

|  | Niet | In lichte mate | In redelijke mate | In behoorlijke mate | In sterke mate |
| --- | --- | --- | --- | --- | --- |
| Als kind werd ik door mijn ouders of leraren gevoelig of verlegen gevonden. | 0 | 1 | 2 | 3 | 4 |
| Ik heb het gevoel dat ik dingen misloop doordat ik niet snel een keuze kan maken. | 0 | 1 | 2 | 3 | 4 |
| Ik heb de neiging om lang opgewonden of ontroerd te blijven nadat ik een goede film heb gezien. | 0 | 1 | 2 | 3 | 4 |
| Wanneer ik in het openbaar ben voel ik me overweldigd, omdat ik niet alles in de gaten kan houden wat er om me heen gebeurt. | 0 | 1 | 2 | 3 | 4 |
| Ik merk dat ik een menigte of de omgeving in de gaten moet houden wanneer ik in het openbaar of op een nieuwe plek ben. | 0 | 1 | 2 | 3 | 4 |
| Ik merk dat ik nogal licht geraakt ben. | 0 | 1 | 2 | 3 | 4 |
| Ik schrik gemakkelijk. | 0 | 1 | 2 | 3 | 4 |
| Ik denk dat het ergste zal gebeuren. | 0 | 1 | 2 | 3 | 4 |
| Ik raak snel geïrriteerd. | 0 | 1 | 2 | 3 | 4 |
| Ik ben nogal gevoelig voor pijn. | 0 | 1 | 2 | 3 | 4 |
| Mijn gezicht voelt warm. | 0 | 1 | 2 | 3 | 4 |
| Mijn armen en benen voelen stijf. | 0 | 1 | 2 | 3 | 4 |
| Zweten bij angst (niet door warmte). | 0 | 1 | 2 | 3 | 4 |
| Het warm hebben bij angst. | 0 | 1 | 2 | 3 | 4 |
| Een snellere of diepere ademhaling. | 0 | 1 | 2 | 3 | 4 |
| Benauwd voelen rond of in de borst. | 0 | 1 | 2 | 3 | 4 |
| Bonzen van het hart. | 0 | 1 | 2 | 3 | 4 |
| Tintelingen in de vingers. | 0 | 1 | 2 | 3 | 4 |
| Bang | 0 | 1 | 2 | 3 | 4 |
| Prikkelbaar | 0 | 1 | 2 | 3 | 4 |
| Vijandig | 0 | 1 | 2 | 3 | 4 |
| Ik word vaak gekweld door schuldgevoelens. | 0 | 1 | 2 | 3 | 4 |
| Ik val moeilijk in slaap. | 0 | 1 | 2 | 3 | 4 |
| Ik heb moeite met doorslapen. | 0 | 1 | 2 | 3 | 4 |
| Zweethanden of zweten van andere lichaamsdelen als ik in mijn bed lig en wil slapen. | 0 | 1 | 2 | 3 | 4 |
| De gebeurtenissen van de dag overdenken of in mijn hoofd herhalen als ik in bed lig en wil slapen. | 0 | 1 | 2 | 3 | 4 |
| Mentaal actief en alert zijn als ik in mijn bed lig en wil slapen. | 0 | 1 | 2 | 3 | 4 |

THDQ scores:

*De score van elke dimensie wordt berekend door het gemiddelde te nemen van de items die bij de dimensie horen.*

Anxious 2, 8, 19, 22

Somatic 12, 15, 16, 17, 18

Sensitive 1, 3, 7, 10

Sleep-related 23, 24, 26, 27

Irritable 6, 9, 20, 21

Vigilant 4, 5

Sudomotor 11, 13, 14, 25

**Transdiagnostic hyperarousal dimensions questionnaire (EN)**

Below you will see a list of words and phrases. People differ in how much these words or phrases describe how they, in general, experience themselves. Please indicate for each word or phrase to what extent it applies to you personally.

|  | Not | Slightly | Moderately | Considerably | Strongly |
| --- | --- | --- | --- | --- | --- |
| When I was a child, my parents or teachers considered me as sensitive or shy. | 0 | 1 | 2 | 3 | 4 |
| I feel like I am missing out on things because I cannot make up my mind soon enough. | 0 | 1 | 2 | 3 | 4 |
| I tend to remain excited or moved for a long period of time after seeing a good movie. | 0 | 1 | 2 | 3 | 4 |
| When I am in public, I feel overwhelmed because I cannot keep track of everything going on around me. | 0 | 1 | 2 | 3 | 4 |
| I notice that when I am in public or new places, I need to scan the crowd or surroundings. | 0 | 1 | 2 | 3 | 4 |
| I notice that I’m agitated | 0 | 1 | 2 | 3 | 4 |
| I startle easily. | 0 | 1 | 2 | 3 | 4 |
| I think that the worst will happen. | 0 | 1 | 2 | 3 | 4 |
| I am easily irritated | 0 | 1 | 2 | 3 | 4 |
| I tend to be more sensitive to pain. | 0 | 1 | 2 | 3 | 4 |
| My face feels hot. | 0 | 1 | 2 | 3 | 4 |
| My arms and legs feel stiff. | 0 | 1 | 2 | 3 | 4 |
| Hot/cold sweats when anxious (unrelated to warmth) | 0 | 1 | 2 | 3 | 4 |
| Feeling hot when anxious. | 0 | 1 | 2 | 3 | 4 |
| Faster or deeper breathing | 0 | 1 | 2 | 3 | 4 |
| Tight feelings in the chest | 0 | 1 | 2 | 3 | 4 |
| Palpitations | 0 | 1 | 2 | 3 | 4 |
| Tingling fingers | 0 | 1 | 2 | 3 | 4 |
| Afraid | 0 | 1 | 2 | 3 | 4 |
| Irritable | 0 | 1 | 2 | 3 | 4 |
| Hostile | 0 | 1 | 2 | 3 | 4 |
| I am often troubled about feelings of guilt | 0 | 1 | 2 | 3 | 4 |
| I have trouble falling asleep. | 0 | 1 | 2 | 3 | 4 |
| I have trouble staying asleep. | 0 | 1 | 2 | 3 | 4 |
| I experience sweating of my hands or other parts of my body when I'm in bed to sleep. | 0 | 1 | 2 | 3 | 4 |
| I review or ponder events of the day when I'm in bed to sleep. | 0 | 1 | 2 | 3 | 4 |
| I am mentally alert and active when I'm in bed to sleep. | 0 | 1 | 2 | 3 | 4 |

THDQ scores:

*The score for each dimension is calculated by taking the average of the items associated with that dimension.*

Anxious 2, 8, 19, 22

Somatic 12, 15, 16, 17, 18

Sensitive 1, 3, 7, 10

Sleep-related 23, 24, 26, 27

Irritable 6, 9, 20, 21

Vigilant 4, 5

Sudomotor 11, 13, 14, 25

**Transdiagnostic hyperarousal dimensions questionnaire (DU)**

Im Folgenden finden Sie eine Liste von Wörtern und Sätzen. Die Menschen unterscheiden sich darin, wie sehr diese Wörter oder Sätze beschreiben, wie sie sich im Allgemeinen erleben. Bitte geben Sie für jedes Wort oder jeden Satz an, inwieweit er auf Sie persönlich zutrifft.

|  | Trifft nicht zu | Trifft etwas zu | Trifft mässig zu | Trifft erheblich zu | Trifft stark zu |
| --- | --- | --- | --- | --- | --- |
| Als Kind wurde ich von meinen Eltern oder Lehrern als sensibel oder schüchtern angesehen. | 0 | 1 | 2 | 3 | 4 |
| Ich habe das Gefühl, Dinge zu verpassen, weil ich mich nicht schnell genug entscheiden kann. | 0 | 1 | 2 | 3 | 4 |
| Ich neige dazu, nach einem guten Film noch lange Zeit begeistert oder bewegt zu sein. | 0 | 1 | 2 | 3 | 4 |
| Wenn ich in der Öffentlichkeit bin, fühle ich mich überfordert, weil ich nicht alles mitbekomme, was um mich herum passiert. | 0 | 1 | 2 | 3 | 4 |
| Ich merke, dass ich, wenn ich in der Öffentlichkeit oder an neuen Orten bin, die Menge oder die Umgebung scannen muss. | 0 | 1 | 2 | 3 | 4 |
| Ich merke, dass ich aufgewühlt bin | 0 | 1 | 2 | 3 | 4 |
| Ich erschrecke leicht. | 0 | 1 | 2 | 3 | 4 |
| Ich denke, dass das Schlimmste passieren wird. | 0 | 1 | 2 | 3 | 4 |
| Ich bin leicht reizbar | 0 | 1 | 2 | 3 | 4 |
| Ich neige dazu, schmerzempfindlich zu sein. | 0 | 1 | 2 | 3 | 4 |
| Mein Gesicht fühlt sich heiß an. | 0 | 1 | 2 | 3 | 4 |
| Meine Arme und Beine fühlen sich steif an. | 0 | 1 | 2 | 3 | 4 |
| Heiße/kalte Schweißausbrüche bei Unruhe (unabhängig von der Wärme) | 0 | 1 | 2 | 3 | 4 |
| Heißes Gefühl, wenn man Angst hat. | 0 | 1 | 2 | 3 | 4 |
| Schnellere oder tiefere Atmung | 0 | 1 | 2 | 3 | 4 |
| Brustschmerzen | 0 | 1 | 2 | 3 | 4 |
| Herzrasen | 0 | 1 | 2 | 3 | 4 |
| Taubheitsgefühl oder Kribbeln in den Fingern oder Zehen | 0 | 1 | 2 | 3 | 4 |
| Ängstlich | 0 | 1 | 2 | 3 | 4 |
| Gereizt | 0 | 1 | 2 | 3 | 4 |
| Feindselig | 0 | 1 | 2 | 3 | 4 |
| Ich bin oft von Schuldgefühlen geplagt | 0 | 1 | 2 | 3 | 4 |
| Ich habe Probleme beim Einschlafen | 0 | 1 | 2 | 3 | 4 |
| Ich habe Schwierigkeiten durchzuschlafen. | 0 | 1 | 2 | 3 | 4 |
| Ich schwitze an den Händen oder an anderen Körperteilen, wenn ich im Bett liege, um zu schlafen. | 0 | 1 | 2 | 3 | 4 |
| Ich lasse die Ereignisse des Tages Revue passieren oder denke darüber nach, wenn ich im Bett liege, um zu schlafen. | 0 | 1 | 2 | 3 | 4 |
| Ich bin geistig wach und aktiv, wenn ich im Bett liege, um zu schlafen. | 0 | 1 | 2 | 3 | 4 |

THDQ scores:

*Die Punktzahl jeder Dimension wird berechnet, indem der Durchschnitt der zu dieser Dimension gehörenden Items ermittelt wird.*

Anxious 2, 8, 19, 22

Somatic 12, 15, 16, 17, 18

Sensitive 1, 3, 7, 10

Sleep-related 23, 24, 26, 27

Irritable 6, 9, 20, 21

Vigilant 4, 5

Sudomotor 11, 13, 14, 25
