# Supplementary material for: Hyperarousal transdiagnostically dissected: different dimensions characterize mood, anxiety, insomnia, posttraumatic stress and attention deficit hyperactivity disorders
Source: eClinicalMedicine. 2026 Mar 12;94:103810. doi: 10.1016/j.eclinm.2026.103810 (PMC13133538; doi:10.1016/j.eclinm.2026.103810)
Supplement: Translated Abstract [file mmc2.docx]

*The following translations in Dutch were submitted by the authors and we reproduce them as supplied. They have not been peer reviewed. Our editorial processes have only been applied to the original abstract in English, which should serve as reference for this manuscript.*

**Achtergrond:** Hyperarousal is een veelvoorkomend symptoom dat bepalend is voor de ernst van slapeloosheid, depressie, angst, posttraumatische stress stoornis en ADHD. Hyperarousal is echter een losjes gedefinieerd concept, dat met per stoornis verschillende vragenlijsten wordt gemeten. Deze studie onderzocht of hyperarousal één gemeenschappelijk transdiagnostisch concept is of juist meerdere, mogelijk stoornis-specifieke, dimensies heeft.

**Methoden:** In dit cohortonderzoek werden deelnemers tussen december 2023 en juni 2024 geworven via de media en het Nederlands Slaapregister. We hebben 467 volwassenen (gemiddelde leeftijd 58,3 jaar [bereik 21-89]; 77,6% vrouw) geïncludeerd met een breed scala aan psychiatrische diagnoses en verschillende niveaus van ernst van symptomen. Deelnemers vulden online vragenlijsten over hyperarousal en de ernst van stoornissymptomen (Insomnia Severity Index, Rapid Measurement Toolkit-20 en ADHD Self-Report Scale) in. Factoranalyses evalueerden mogelijke dimensies in hyperarousal aan de hand van 221 items uit 18 vragenlijsten. Met multiple regressiemodellen werden profielen gemaakt van de meest relevante hyperarousal-dimensies voor de ernst van de symptomen van insomnia, depressie, gegeneraliseerde angststoornis, sociale angststoornis, paniekstoornis, posttraumatische stressstoornis en ADHD. We selecteerden 27 items om de zeven dimensies van hyperarousal te kunnen meten met een korte Transdiagnostic Hyperarousal Dimensions Questionnaire (THDQ). Tussen maart 2025 en april 2025 werd een tweede steekproef gerekruteerd die de THDQ invulden voor confirmatieve factor analyse en verdere validatie (n = 592; gemiddelde leeftijd 61,0 jaar [bereik 19-89]; 65,2% vrouw). Om te evalueren of dimensie scores te schatten zijn met behulp van bestaande UK Biobank-items, berekenden we de polychorische correlaties tussen de 27 geselecteerde items en 22 UK Biobank-items, beschikbaar voor 467 deelnemers.

**Bevindingen:** Exploratieve factoranalyse identificeerde 7 dimensies, die 50,2% van de variantie verklaarden. Ze vertegenwoordigen angstige-, somatische-, sensitieve-, slaapgerelateerde-, prikkelbare-, waakzame- en transpiratie-gerelateerde hyperarousal. Multiple regressiemodellen lieten zien dat hyperarousal-dimensies in verschillende mate samenhangen met de ernst van slapeloosheid, depressie, angst, paniek, posttraumatische stress en symptomen van ADHD (gestandaardiseerde bèta-coëfficiënten = -.10-0,70). Vervolgens ontwikkelden en valideerden we de THDQ die met 27 items elke dimensie betrouwbaar kon meten (CFI = 0,92, RMSEA = 0,05, Cronbach's alpha = 0,90). Ten slotte toonden we aan dat items van de UK Biobank een schatting kunnen geven van angstige-, prikkelbare- en slaapgerelateerde hyperarousal (r = 0,75-0,85).

**Interpretatie:** Het meten van verschillende dimensies van hyperarousal met de THDQ kan begrip van hyperarousal bevorderen en mogelijk aanwijzingen geven voor een betere behandeling van meerdere psychische stoornissen.

**Financiering:** European Research Council (ERC) en ZonMw, de samenwerking tussen ZorgOnderzoek Nederland (ZON) en het gebied Medische Wetenschappen (MW) van de Nederlandse Organisatie voor Wetenschappelijk Onderzoek (NWO).

**Trefwoorden:** Hyperarousal, Transdiagnostisch, Slapeloosheid, Angststoornissen, Posttraumatische stressstoornis, Depressie
